# PRIME: an interpretable artificial intelligence model based on liquid biopsy improves prediction of progression risk in non-small cell lung cancer

**DOI:** 10.1186/s40779-025-00679-z

**Published:** 2026-01-06

**Authors:** Yu Wang, Yong-Bo Xiang, Xiao-Wei Chen, Tao Zhang, Jian-Yang Wang, Wen-Yang Liu, Lei Deng, Lu-Hua Wang, Shu-Geng Gao, Nan Bi

**Affiliations:** 1 Department of Radiation Oncology, National Cancer Center/National Clinical Research Center for Cancer/Cancer Hospital, Chinese Academy of Medical Sciences and Peking Union Medical College, Beijing, 100021 China; 2 Department of Thoracic Surgery, National Cancer Center/National Clinical Research Center for Cancer/Cancer Hospital, Chinese Academy of Medical Sciences and Peking Union Medical College, Beijing, 100021 China; 3 Department of Radiation Oncology, National Cancer Center/National Clinical Research Center for Cancer/Cancer Hospital & Shenzhen Hospital, Chinese Academy of Medical Sciences and Peking Union Medical College, Shenzhen, 518116 Guangdong China; 4 Key Laboratory of Minimally Invasive Therapy Research for Lung Cancer, Chinese Academy of Medical Sciences, Beijing, 100021 China; 5 State Key Laboratory of Molecular Oncology, National Cancer Center/National Clinical Research Center for Cancer/Cancer Hospital, Chinese Academy of Medical Sciences and Peking Union Medical College, Beijing, 100021 China

**Keywords:** Non-small cell lung cancer, Artificial intelligence, Liquid biopsy, Machine learning, Circulating tumor DNA, Minimal residual disease

## Abstract

**Background:**

Despite the predictive impact of circulating tumor DNA (ctDNA) minimal residual disease (MRD), accurate prediction of failure risk after curative-intent treatments for early-stage or localized non-small cell lung cancer (NSCLC) patients to guide personalized therapy remains challenging. This study aimed to develop and validate an interpretable artificial intelligence-assisted model using global data resources.

**Methods:**

Liquid biopsy data, blood-based genomic alterations, clinicopathological features, and survival outcomes of stage I–III NSCLC patients who underwent surgery or definitive chemoradiotherapy were collected from 6 cohorts. PRIME (Progression Risk prediction by Interpretable Machine learning on ctDNA-MRD, Mutations, and clinical-therapeutic features) was trained by 6 machine learning algorithms across 4 cohorts and validated in 2 independent cohorts. Model performance was evaluated by the area under the curve (AUC) and interpreted by SHapley Additive exPlanations (SHAP). Whole-exome sequencing (WES) or whole-genome sequencing (WGS) of tumor tissue from 430 stage II–III NSCLC patients and RNA-sequencing (RNA-seq) data from 1149 subjects, sourced from The Cancer Genome Atlas, were used to validate the prognostic effect of mutations identified in peripheral blood and investigate the underlying mechanisms.

**Results:**

A global dataset encompassing 781 blood samples from 493 patients was analyzed. Clinical stage, pre-treatment ctDNA, post-treatment MRD, blood-based Kelch-like ECH-associated protein 1 (*KEAP1*), serine/threonine kinase 11 (*STK11*), and cyclin-dependent kinase inhibitor 2A (*CDKN2A*) mutations, and treatment modality were significantly associated with the risk of disease progression and were thereby included in the model training. WES/WGS and RNA-seq confirmed the poor prognostic effect of *KEAP1*, *STK11*, and *CDKN2A* mutations, which were characterized by the suppressive tumor microenvironment and attenuated humoral immunity. The neural network (NN) model exhibited optimal prediction of treatment failure risk in the training (AUC = 0.85, 95% CI 0.81–0.89) and validation sets (AUC = 0.82, 95% CI 0.74–0.89). SHAP analysis indicated that MRD (+0.306), treatment modality (+0.128), and pre-treatment ctDNA (+0.043) ranked in the top 3 contributions. NN-PRIME outperformed single liquid biopsy biomarkers and clinical-therapeutic signatures, and demonstrated consistent robustness across different clinical scenarios. High-risk patients identified by NN-PRIME had poorer prognoses but derived significant benefits from adjuvant therapy after surgery.

**Conclusions:**

As an interpretable model integrating readily-accessible and crucial clinical-genomic predictors, PRIME achieves enhanced performance, allowing for early outcome prediction, refined risk stratification, and personalized clinical decision-making.

**Supplementary Information:**

The online version contains supplementary material available at 10.1186/s40779-025-00679-z.

## Background

Non-small cell lung cancer (NSCLC) is the leading cause of cancer-related death [1, 2]. Curative-intent treatment strategies for patients with early-stage or localized NSCLC include surgical resection and radical chemoradiotherapy (CRT) [3]. Despite advances in surgical procedures, radiation techniques, and combined therapies with immune checkpoint inhibitors (ICIs), patient outcomes remain poor, with a high recurrence rate ranging from 30% to 55% [4, 5]. Therefore, there is an urgent need for effective predictive models that can determine the risk of disease progression early, stratify prognosis accurately, and guide personalized escalated or de-escalated adjuvant treatment decisions.

Although radiological surveillance is currently the clinical follow-up for early-stage and localized NSCLC patients after curative treatment, such monitoring tools can identify only macroscopic disease relapse [6]. Given postsurgical anatomical changes as well as indistinguishable radiation pneumonitis from disease progression, radiographic monitoring can be inconclusive [7, 8]. Liquid biopsies, including circulating tumor DNA (ctDNA)-based minimal (or molecular) residual disease (MRD) detection, provide a noninvasive alternative and the ability to predict disease progression early with high specificity (approximately 71–100%) [9–11]. However, the insufficient sensitivity (36–100%) [10] of single ctDNA-MRD detection for predicting recurrence remains an unsolved problem. More than 50% of patients with negative post-treatment MRD will eventually experience disease recurrence [12, 13], highlighting the necessity of improving sensitivity. Recent studies have suggested that integrating ctDNA-based genomic alterations into a multi-parameter model could improve predictive performance and assist with more personalized prediction [14–16].

In addition to incorporating circulating genomic features into combinatorial models, several machine learning (ML) algorithm-based studies using multi-modality data, including clinicopathologic characteristics, treatment regimens, and peripheral blood biomarkers, have shown enhanced prediction effects and promising clinical applicability [17–20]. Compared with single biomarkers or conventional statistical combined models, such as regression or classification tools constructed by combining ctDNA testing with other variables, ML approaches possess the advantages of capturing more nuanced and highly individualized features in the field of biomedicine [21, 22], thereby effectively improving the prediction power [23]. Such comprehensive and informative artificial intelligence (AI)-assisted models are invaluable for non-metastatic NSCLC, since they help identify the failure risk early after local therapies and guide risk-adaptive decisions on adjuvant systemic treatments [24]. Moreover, in contrast to the inexplicable “black box” nature of deep learning algorithms, ML models offer better interpretability and are more suitable for clinical practice [25–27]. However, research in this field is currently lacking.

Currently, in the real-world setting, patients often undergo different ctDNA testing techniques on various platforms, including fixed panels or personalized panels. Although the detection sensitivity and specificity of each platform likely differ, and it is difficult to directly compare results across panels, there remains an urgent need for a comprehensive summary of results obtained from different techniques to assess the overall predictive performance. This will help make an objective assessment of the clinical value of liquid biopsy and guide real-world medical practice. This study aimed to develop a novel model named PRIME (Progression Risk prediction using Interpretable ML on ctDNA-based genomic Mutations, MRD, and clinical-therapeutic features) for early prediction of treatment failure risk and clinical outcomes in early-stage and localized NSCLC patients.

## Methods

### Description of the data and participants

**Fig. 1:**
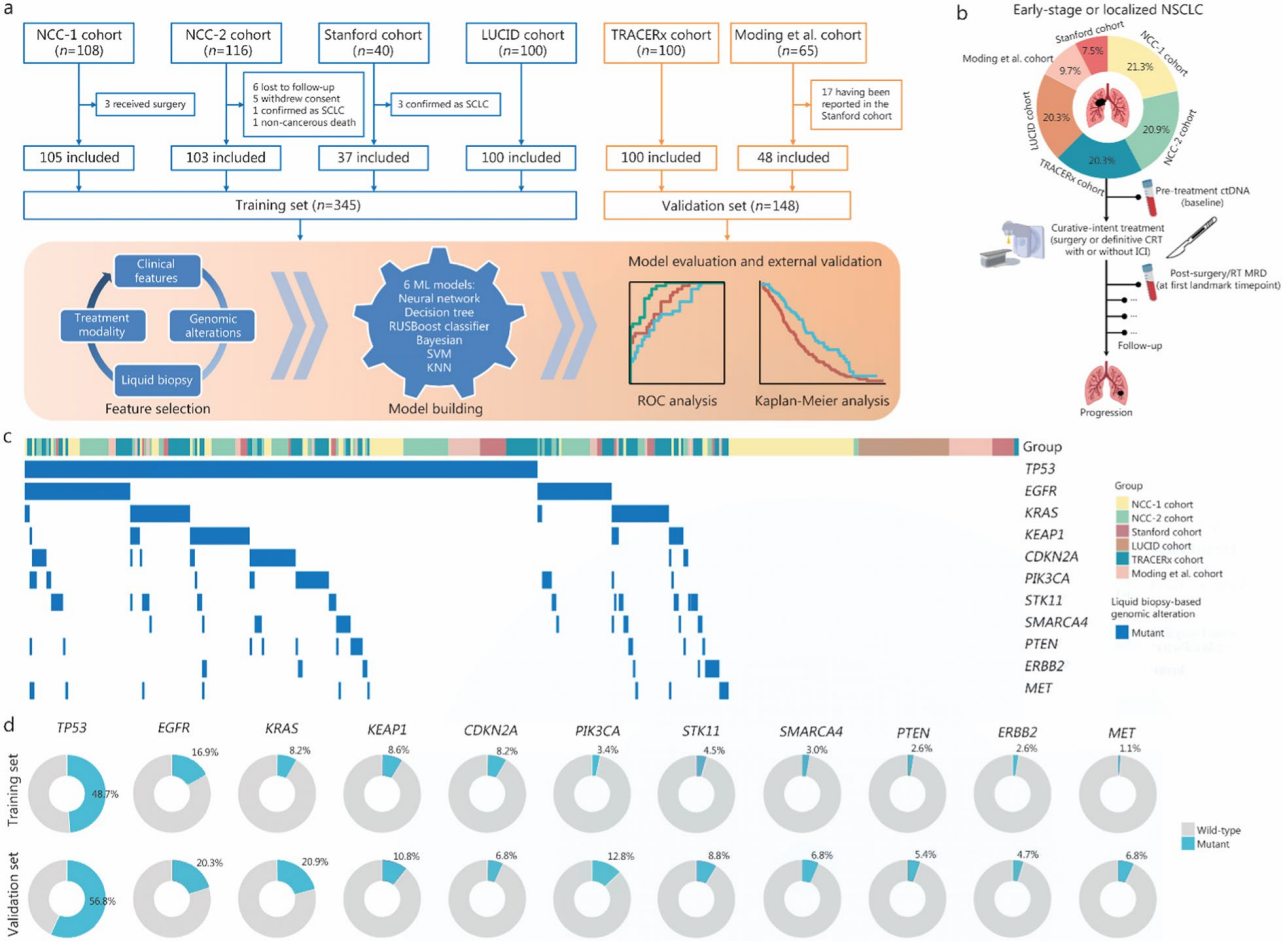
Study overview. **a** Description of study procedure. **b** Flowchart of peripheral blood sample collection. **c** Heatmap plot of pre-treatment ctDNA-based tumor mutations for patients with variants identified in tumor tissue or plasma (*n* = 415). **d** Donut plots displaying the distribution of identified ctDNA-based mutations in the training set (*n* = 267) and validation set (*n* = 148). A total of 78 patients in the training set with unavailable blood-based mutation sequencing data before treatment. NSCLC non-small cell lung cancer, SCLC small cell lung cancer, ML machine learning, RUSBoost random under-sampling boosting, SVM support vector machine, KNN K-nearest neighbors, AUC area under the curve, ctDNA circulating tumor DNA, CRT chemoradiotherapy, ICI immune checkpoint inhibitor, RT radiotherapy, MRD minimal residual disease, NCC National Cancer Center, LUCID LUng cancer Circulating tumour DNA, TRACERx Tracking Non-Small-Cell Lung Cancer Evolution Through Therapy

This study analyzed data from 6 cohorts (Fig. 1a). NCC-1 and NCC-2 cohorts were in-house datasets from National Cancer Center (Beijing, China), comprising 105 unresectable and 103 resectable stage I–III NSCLC patients, respectively, treated between 2018 and 2022. Patients in NCC-1 cohort received definitive CRT with or without consolidation ICI therapy, and those in NCC-2 cohort underwent radical surgery. Longitudinal ctDNA profiling was performed at pre-treatment and post-treatment timepoints in both cohorts. LUCID (LUng cancer Circulating tumour DNA) [28], TRACERx (Tracking Non-Small-Cell Lung Cancer Evolution Through Therapy) [29, 30], Stanford [31], and Moding et al. [32] cohorts were available open-source datasets that, at the time this study was conducted, met the following inclusion criteria: 1) early-stage or localized NSCLC patients with survival outcomes; 2) curative-intent treatments including surgery or definitive CRT; and 3) longitudinal ctDNA profiling before and after therapy. Among the 6 cohorts, 2 used tumor-informed personalized ctDNA panels (LUCID [28] and TRACERx [29, 30]), and 4 employed fixed-panel ctDNA detection (NCC-1 and NCC-2, Stanford [31], and Moding et al. [32]). Considering the heterogeneity of sequencing platforms used by patients in the real world, we endeavored to incorporate data from different ctDNA testing techniques into the model training, and assessed the predictive performance of the final integrated PRIME model in different clinical contexts, aiming to guide real-world medical practice. For the enrolled patients, we collected their clinical characteristics, treatment regimens, survival outcomes, and ctDNA testing results, including pre-treatment ctDNA at baseline and post-treatment ctDNA-MRD detection at the first landmark time point, that is, within 2 weeks to 4 months after radical treatment ended (Fig. 1b). The baseline information of all the subjects is summarized in Additional file 1: Table S1. For details regarding the clinical procedures, patient eligibility criteria, and ctDNA sequencing methods used in each cohort, please refer to the Additional file 2: Methods. This study was approved by the Ethics Committee of Chinese Academy of Medical Sciences (20/453–2649).

### Genomic mutation analyses

As mentioned above, ctDNA-based genotyping involves targeted sequencing of pre-treatment blood samples using fixed panels or personalized panels, which is highly consistent with the clinical utility of liquid biopsy in real-world settings. To validate the prognostic effect of genomic mutations identified by liquid biopsies, we employed whole-exome sequencing (WES) or whole-genome sequencing (WGS) data on tumor tissue samples from 430 patients with stage II–III lung squamous cell carcinoma (LUSC) or lung adenocarcinoma (LUAD) who underwent local therapy. RNA-sequencing (RNA-seq) data from 1149 subjects, including 1041 LUSC or LUAD patients and 108 healthy controls, were used to investigate the underlying mechanisms. WES/WGS and RNA-seq data were retrieved from The Cancer Genome Atlas (TCGA) portal (https://portal.gdc.cancer.gov/). We performed differential gene expression, functional enrichment, and immune infiltration analyses. Heatmaps and supervised clustering were generated by differential expression of preselected genes. All the samples were *Z* score normalized and clustered using Euclidean distance. Gene Ontology (GO) and Kyoto Encyclopedia of Genes and Genomes (KEGG) pathway analyses were used for functional annotations. Gene set enrichment analysis (GSEA) was further conducted.

### Clinical outcome measures

The primary outcome measure of this study was disease progression, defined as disease recurrence after surgery or progressive disease after radiotherapy, which was determined according to Response Evaluation Criteria in Solid Tumors (RECIST) version 1.1 [33]. In this study, we consistently used “progression” to refer to the occurrence of progressive disease in patients treated with definitive radiotherapy and disease recurrence in patients who underwent surgery. For patients who received radical surgical resection (no measurable residual disease after surgery), the secondary outcome was disease-free survival (DFS), defined as the time from diagnosis to the first occurrence of newly measurable local–regional lesions, distant metastasis, or death without prior disease progression [34]. For those treated with definitive radiotherapy (residual measurable disease after treatment), the secondary outcome was progression-free survival (PFS), defined as the time from diagnosis to ≥ 20% growth of local–regional target lesions (according to RECIST version 1.1), distant metastasis, or death without prior progression [34]. In this study, when analyzing the overall population, we consistently used “PFS” to refer to the time to treatment failure (local–regional failure or distant metastasis) or death without treatment failure. The exploratory outcome was overall survival (OS), defined as the time to death from any cause. Clinical outcomes and treatment efficacy were evaluated via radiological examinations.

### Feature selection for the ML models

Feature screening was performed in the training set. Logistic regression analysis was applied to evaluate the effects of clinicopathological characteristics, liquid biopsy biomarkers, ctDNA-based genomic alterations, curative treatment regimens on disease progression (the primary outcome measure). Specifically, features with a *P*-value < 0.05 in logistic regression analysis were considered to be significantly associated with disease progression, and were retained for subsequent ML model construction. The variance inflation factor (VIF) was calculated to assess multicollinearity among features.

### ML model training and validation

The NCC-1 and -2, Stanford, and LUCID cohorts were employed to train the ML models. The TRACERx and Moding et al. cohorts were used for external independent validation. A total of 6 well-established classification learning models were trained and evaluated, including a neural network (NN), decision tree, random under-sampling boosting (RUSBoost) classifier, naive Bayes, support vector machine (SVM), and K-nearest neighbors (KNN). The predictive performance of the model for disease progression risk was assessed using receiver operating characteristic (ROC) curves and area under the curves (AUCs). DeLong’s test was employed to compare the predictive performance of different models and to test for statistical differences in AUCs of ROC curves [35, 36]. We also used F1-score, accuracy, precision, and recall to evaluate model performance. The handling of missing values included deletion or multiple imputation. Missing data were summarized variable-by-variable. When the missingness was < 5%, complete-case analysis was performed. For variables with ≥ 5% missing values deemed missing at random (MAR) or missing completely at random (MCAR), we performed multiple imputation using the multivariate imputation by chained equation for NN. To visually assess whether variables were MAR, MCAR, or missing not at random (MNAR), we used Sankey diagrams to contrast the flow of complete vs. missing data across relevant variable strata. Sensitivity analyses were conducted after excluding individuals with missing data (complete-case analysis) to examine the robustness of the model. SHapley Additive exPlanations (SHAP) were used to interpret the importance and contribution of each feature in the model prediction.

### Standardized ctDNA-MRD detection across cohorts

To minimize the bias across cohorts and ensure consistency and comparability, we extracted the parameters shared across all cohorts and processed them using the same pipeline. All the fixed-panel sequencing platforms used covered the genes of interest in this study, including tumor protein p53 (*TP53*), epidermal growth factor receptor (*EGFR*), Kirsten rat sarcoma viral oncogene homolog (*KRAS*), Kelch-like ECH-associated protein 1 (*KEAP1*), cyclin-dependent kinase inhibitor 2a (*CDKN2A*), phosphatidylinositol-4,5-bisphosphate 3-kinase catalytic subunit alpha (*PIK3CA*), serine/threonine kinase 11 (*STK11*), SWI/SNF-related, matrix-associated, actin-dependent regulator of chromatin, subfamily A, member 4 (*SMARCA4*), phosphatase and tensin homolog (*PTEN*), Erb-B2 receptor tyrosine kinase 2 (*ERBB2*), and mesenchymal-epithelial transition factor (*MET*), which are canonical NSCLC driver mutations [37, 38]. To pool available individual-level data from different cohorts, we also aligned their MRD calling pipelines. A tumor-informed consensus definition was derived by taking the intersection of the technical parameters explicitly documented in every protocol. Consequently, a post-treatment plasma sample was classified as MRD-positive only when all of the following criteria were satisfied: 1) sequencing was restricted to somatic single-nucleotide variants (SNVs) pre-identified in the tumor samples or baseline plasma, with germline and clonal-hematopoiesis variants removed; 2) at least 2 independent SNVs from this patient-specific panel were detected in the same blood draw; 3) each variant exhibited an allele frequency ≥ 0.01% after error-suppression and background polishing; and 4) the cumulative confidence score for the variant set exceeded the study-specific threshold corresponding to ≥ 98% specificity in matched negative-control plasmas. The MRD-landmark time point was defined as the first post-curative sample collected between 2 weeks and 4 months after completion of radical surgery or radiotherapy. This minimum-common-specification preserves the highest shared analytical specificity and allows harmonized downstream analyses.

Additionally, to address the potential bias arising from heterogeneity across different ctDNA detection techniques, we employed a generalized linear mixed-effects model (GLMM). Specifically, we included ctDNA testing panel as a random intercept term, allowing the baseline event probability to vary by technique. This approach provides adjusted effect estimates for the predictor of interest while controlling for between-panel variability. The significance of the panel-level variance component was evaluated using a likelihood ratio test comparing the mixed-effects model to a standard logistic regression model without random effects.

### Statistical analysis

Continuous variables were summarized as mean ± standard deviation, and categorical variables were described as *n* (%). Survival curves were estimated with the Kaplan–Meier method and compared by the log-rank test. For survival analyses, only patients with complete time-to-event data were included. All Kaplan–Meier plots report the exact numbers at risk. Hazard ratios (*HR*s) and 95% confidence intervals (CIs) were calculated by Cox proportional hazard regression modeling. The effects of the variables on disease progression were quantified using logistic regression and presented as *OR*s and 95% CIs. Variables with *P* < 0.05 in logistic regression models were selected for further development of ML models. The distributions of continuous variables were compared by the Wilcoxon signed-rank test. The proportions of categorical data across groups were compared using the Chi-square test or Fisher’s exact test as appropriate. Bonferroni correction was applied for multiple testing correction. Potential correlations were determined using the Spearman and Pearson methods and represented by correlation coefficients (*r*). *P*-values < 0.05 were considered statistically significant. Statistics were analyzed with R software (v4.4.1). ML algorithms and model development processes were implemented with Python (v3.12) and MATLAB (R2022a).

## Results

### Study overview

We compiled a large-scale, global dataset of 781 blood samples from 493 early-stage or localized NSCLC patients treated with curative intent (Fig. 1a), including the training set (*n* = 345) and the validation set (*n* = 148). The clinical characteristics are summarized in Additional file 1: Table S1. Detailed baseline data for patients from each cohort are provided in Additional file 1: Tables S2-S7. The mutational data of 2 proprietary datasets (NCC-1 and NCC-2 cohorts) are presented in Additional file 1: Tables S8-S9.

For eligible participants, pre- and post-treatment peripheral blood samples were collected before and after curative treatments, including surgery or definitive CRT with or without consolidation ICI therapy (Fig. 1b). Among 415 (84.2%) patients with pre-treatment ctDNA variant data, *TP53* (51.6%), *EGFR* (18.1%), *KRAS* (12.8%), *KEAP1* (9.4%), *CDKN2A* (7.7%), *PIK3CA* (6.7%), *STK11* (6.0%), and *SMARCA4* (4.3%) were the most frequently detected candidate driver mutations (Fig. 1c), in accordance with the expected pattern in canonical NSCLC drivers [37, 38]. In the training and validation sets, the frequency of detected alterations remained largely consistent (Fig. 1d). Comparisons of the clinical characteristics between the training and validation datasets are presented in Additional file 1: Table S10. There were no significant differences in age, sex, histopathological type, or pre-treatment ctDNA status between the two groups of patients. However, compared with the training set, the validation set included more patients with stage I disease who received surgery and who were positive for post-treatment MRD (Additional file 1: Table S10). In terms of survival outcomes, after a median follow-up of 36.7 months, the median PFS for all patients was 28.6 months (95% CI 24.4–52.0), and the median OS was 60.0 months [95% CI 60.0–not reached (NR); Additional file 2: Fig. S1].

### Comprehensive features identified for model construction

**Fig. 2:**
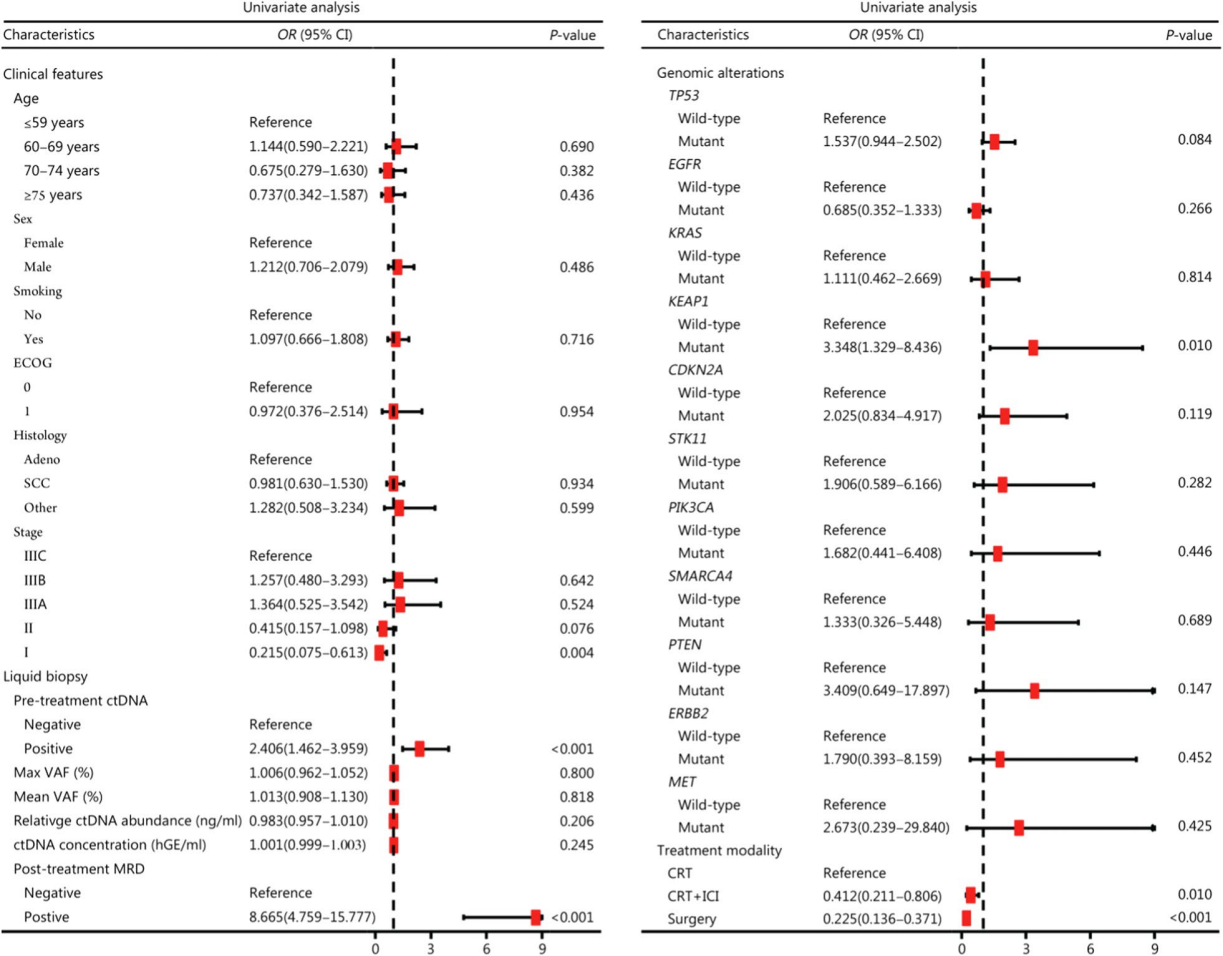
Forest plot of potential predictors for disease progression in the training set (*n* = 345). *OR* odds ratio, CI confidence interval, ECOG Eastern Cooperative Oncology Group, Adeno Adenocarcinoma, SCC squamous cell carcinoma, ctDNA circulating tumor DNA, VAF variant allele frequency, MRD minimal residual disease, CRT chemoradiotherapy, ICI immune checkpoint inhibitor

A comprehensive landscape of 24 potential features was analyzed in the training set (*n* = 345), including clinicopathological characteristics, liquid biopsy biomarkers, ctDNA-based genomics, and treatment modalities (Fig. 2). Compared with stage II–III disease, stage I disease was significantly against the risk of progression (*OR* = 0.215, 95% CI 0.075–0.613; *P* = 0.004). Pre-treatment detectable ctDNA was significantly associated with an increased risk of disease progression (*OR* = 2.406, 95% CI 1.462–3.959; *P* < 0.001), and post-treatment MRD showed a stronger positive correlation with progression (*OR* = 8.665, 95% CI 4.759–15.777; *P* < 0.001). *KEAP1* mutation was also positively correlated with disease progression (*OR* = 3.348, 95% CI 1.329–8.436; *P* = 0.010). Regarding curative-intent treatment modalities, compared with CRT alone, both CRT plus consolidation ICI (*OR* = 0.412, 95% CI 0.211–0.806; *P* = 0.010) and surgery (*OR* = 0.225, 95% CI 0.136–0.371; *P* < 0.001) significantly favored freedom from progression. VIF values for variables in the logistic regression analysis are summarized in Additional file 1: Table S11, and no evident multicollinearity was observed among the features.

### *KEAP1*, *STK11*, and *CDKN2A* mutations suggest poor prognosis

**Fig. 3:**
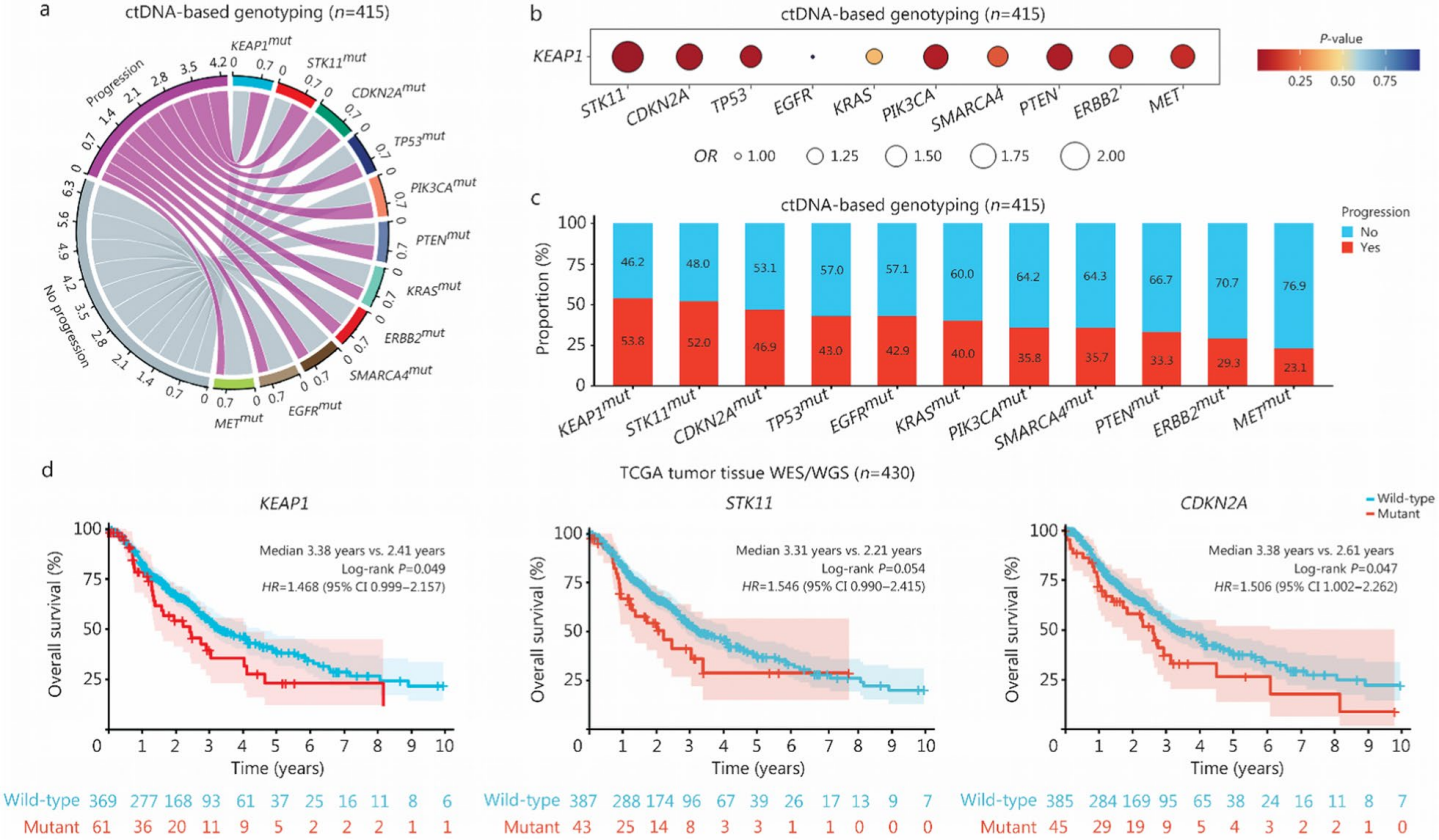
Genomic mutation biomarkers for disease progression. **a** Chord diagram showing *KEAP1*-, *STK11*-, and *CDKN2A*-mutated patients ranking in the top 3 for the proportion of disease progression, among all patients with detectable ctDNA-based variants (*n* = 415). **b** Bubble plots depicting odds ratios (*ORs*) for disease progression conferred by *KEAP1* and co-occurring mutations estimated by logistic regression (*n* = 415). Bubble size indicates the *OR* value, and bubble color reflects the *P* value. **c** Stacked bar plots indicating the proportion of disease progression (red bar) in patients with detectable ctDNA-based variants (*n* = 415). Among all mutations, the overall comparison of progression revealed no significant difference (*P* = 0.292), while the progression rate was significantly higher with *KEAP1*, *STK11*, and *CDKN2A* mutations than with other mutations (Bonferroni-corrected *P* = 0.045). **d** Kaplan–Meier curves of overall survival (OS) stratified by *KEAP1*, *STK11*, and *CDKN2A* mutation status using tumor tissue whole-exome or whole-genome sequencing (WES/WGS) from The Cancer Genome Atlas (TCGA) database. All patients had stage II or III NSCLC and received local treatments (*n* = 430). ctDNA circulating tumor DNA, mut mutant, ns not significant, *HR* hazard ratio, CI confidence interval

We then evaluated the prognostic effects of mutations detected in peripheral blood. Among patients with available pre-treatment ctDNA genotyping data (*n* = 415), disease progression occurred in 53.8%, 52.0%, and 46.9% of patients with *KEAP1*, *STK11*, and *CDKN2A* mutations, respectively, ranking in the top 3 (Fig. 3a). Given that *KEAP1* mutations frequently occur alongside other alterations (co-mutations) [39, 40], we further investigated the effect of co-occurring *KEAP1* and other mutations on disease progression. Consistent with previous research [39–41], we found that *KEAP1*/*STK11* (*OR* = 2.23, 95% CI 1.27–3.91; *P* = 0.005) and *KEAP1*/*CDKN2A* (*OR* = 1.84, 95% CI 1.09–3.11; *P* = 0.023) were associated with an increased risk of progression (Fig. 3b). Among all mutations, the overall comparison of disease progression rate revealed no significant difference (*P* = 0.292; Fig. 3c). However, the proportion of disease progression was significantly higher with *KEAP1*, *STK11*, and *CDKN2A* mutations than with other mutations (Bonferroni-corrected *P* = 0.045; Fig. 3c). The poor prognostic value of *KEAP1*, *STK11*, and *CDKN2A* mutations was further validated in the tumor tissue WES/WGS data. A total of 430 patients from TCGA with stage II–III NSCLC who received local treatment were included in the analysis. Baseline characteristics are displayed in Additional file 1: Table S12. Patients with *KEAP1*-mutated disease had shorter OS (median 2.41 years vs. 3.38 years; *P* = 0.049; Fig. 3d). Similarly, compared with *STK11-* and *CDKN2A-*wild-type patients, those with *STK11* (median 2.21 years vs. 3.31 years; *P* = 0.054) and *CDKN2A* (median 2.61 years vs. 3.38 years; *P* = 0.047; Fig. 3d) mutations were associated with marginally poorer OS. Taken together, baseline *KEAP1*, *STK11*, and *CDKN2A* mutations detected by liquid biopsy indicate a poor prognosis, which was also confirmed by tumor tissue-based mutation sequencing data.

### Transcriptomic profiling and immune landscape of *KEAP1*, *STK11*, and *CDKN2A*

**Fig. 4:**
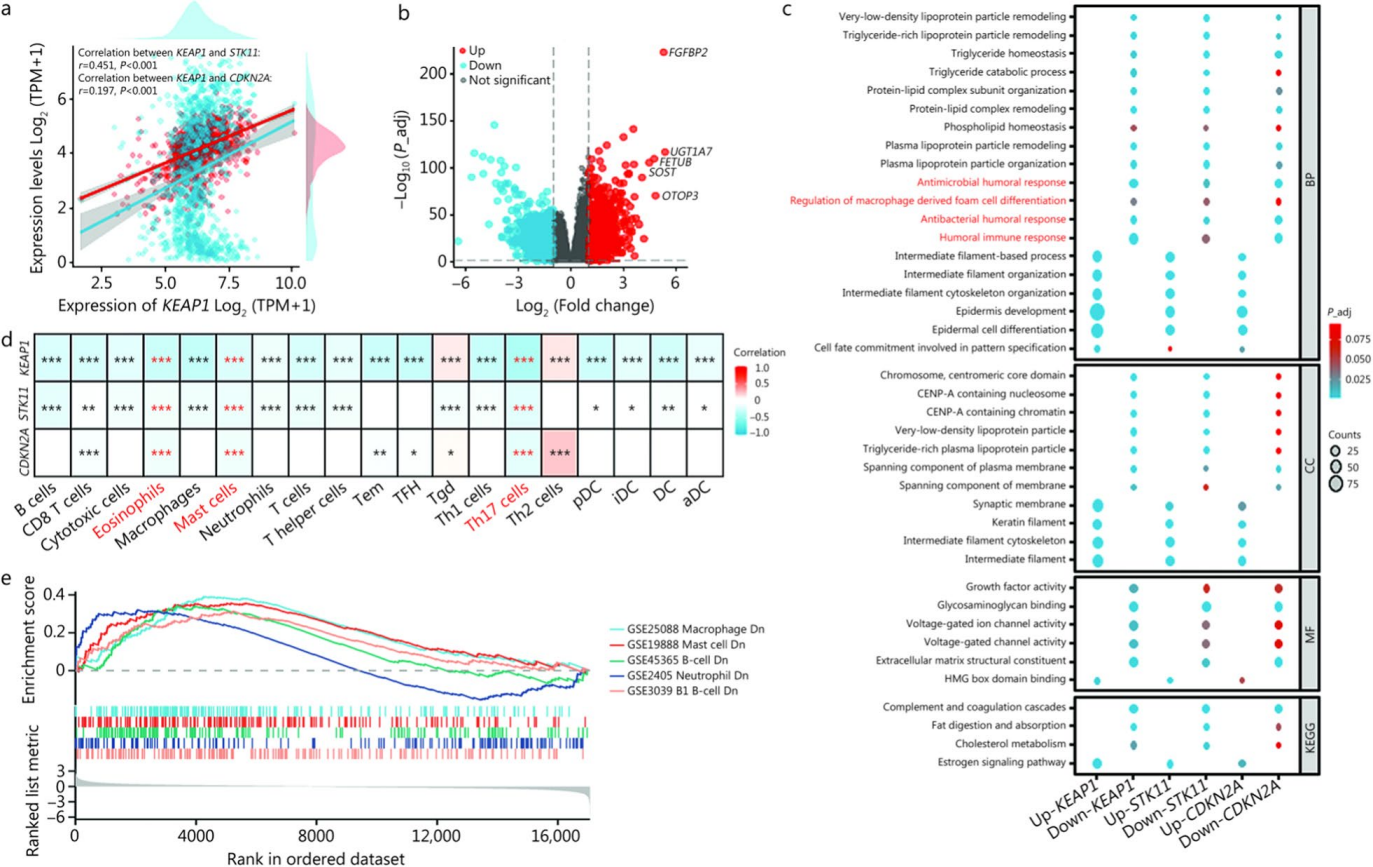
Immune landscape of *KEAP1-*, *STK11-*, and *CDKN2A-*mutant tumors. **a** Scatter plot of Pearson correlation analyses for *KEAP1*, *STK11*, and *CDKN2A*. Blue dots refer to the expression of *KEAP1* and *STK11* genes, and the blue fitted curve indicates a positive correlation (*r* = 0.451). Red dots refer to the expression of *CDKN2A*, and the red fitted curve indicates a negligible correlation between *KEAP1* and *CDKN2A* (*r* = 0.197). **b** Volcano plot displaying differentially expressed genes in *KEAP1*-, *STK11*-, and *CDKN2A*-mutated tumors. **c** Bubble chart illustrating the Gene Ontology/Kyoto Encyclopedia of Genes and Genomes (GO/KEGG) pathway enrichment analysis of down-regulated genes. **d** Heatmap indicating the infiltrating immune cells in the *KEAP1-*, *STK11-*, and *CDKN2A-*mutated tumors. **e** Gene set enrichment analysis (GSEA) plots showing enriched pathways in the down-regulation of immune cells. ^*^P < 0.05, ^**^P < 0.01, ^***^P < 0.001. TPM transcripts per million, BP biological process, CC cellular component, MF molecular function, *P*_adj adjusted *P*-value, Tem T effector memory, TFH T follicular helper, Tgd T gamma delta, Th T helper, pDC plasmacytoid dendritic cell, iDC immature dendritic cell, DC dendritic cell, aDC activated dendritic cell, Dn downregulated, CENP centromere protein, HMG high mobility group

We next explored the transcriptomic data of *KEAP1*, *STK11*, and *CDKN2A* via RNA-seq of 1041 patients with LUSC or LUAD and 108 healthy control subjects. Compared with those in normal tissue, *KEAP1*, *STK11*, and *CDKN2A* were highly expressed in the tumor samples of NSCLC patients (*P* < 0.01; Additional file 2: Fig. S2a). Although the *STK11*, *SMARCA4*, and *KEAP1* genes were clustered together, *CDKN2A* was separated from them and was in the same macrocluster with *EGFR* and *PIK3CA* (Additional file 2: Fig. S2b). Consistently, compared with those of other genes, the correlation coefficients of *CDKN2A* with *KEAP1* (Spearman *r* = 0.183, *P* < 0.001) and *CDKN2A* with *STK11* (Spearman *r* = 0.154, *P* < 0.001) were relatively lower (Additional file 2: Fig. S2c). Pearson correlation analyses revealed that the correlation between *CDKN2A* and *KEAP1* was negligible (Pearson *r* = 0.197, *P* < 0.001), whereas *KEAP1* was positively correlated with *STK11* (Pearson *r* = 0.451, *P* < 0.001; Fig. 4a). Logistic regression suggested that *KEAP1* (*OR* = 2.012, 95% CI 1.036–3.907; *P* = 0.039) and *KEAP1/STK11* (*OR* = 2.229, 95% CI 1.272–3.906; *P* = 0.005) mutations were significantly associated with an increased risk of disease progression (Additional file 2: Fig. S3a). For patients with *KEAP1/STK11* mutations, a higher proportion of progression was observed, compared to wild-type patients (58.2% vs. 35.6%; *P* = 0.004; Additional file 2: Fig. S3b). The above findings suggest that *KEAP1* and *STK11* may have co-mutated expression, while *CDKN2A* does not show a clear correlation pattern with them.

Furthermore, a volcano plot of the differentially expressed genes in *KEAP1/STK11/CDKN2A*-mutated patients is shown in Fig. 4b. The down-regulated genes were significantly enriched in pathways associated with humoral immune responses and macrophage regulation (Fig. 4c). Consistently, immune infiltration analysis revealed striking reductions in various immune cells in *KEAP1-*, *STK11-* or *CDKN2A*-mutated tumors (Additional file 2: Fig. S4), characterizing the suppressive tumor immune microenvironment (TIME). Among them, eosinophils, mast cells, and T helper 17 cells were concomitantly decreased in *KEAP1-*, *STK11-*, and *CDKN2A*-mutated tumors (Fig. 4d). GSEA plots (Fig. 4e) confirmed the downregulation of pathways associated with macrophage differentiation [normalized enrichment score (NES) = −2.026, false discovery rate (FDR) < 0.001], mast-cell activation (NES = −1.851, FDR = 0.006), and B-cell-mediated immunity (NES = -1.755, FDR = 0.026; Additional file 2: Fig. S5), highlighting distinct humoral immunosuppression. Taken together, mutations in *KEAP1*, *STK11*, and *CDKN2A* could collectively drive a suppressive “cold” TIME, attenuate humoral immunity, and confer poor prognosis.

### Multi-modal features effectively predict the risk of disease progression

**Fig. 5:**
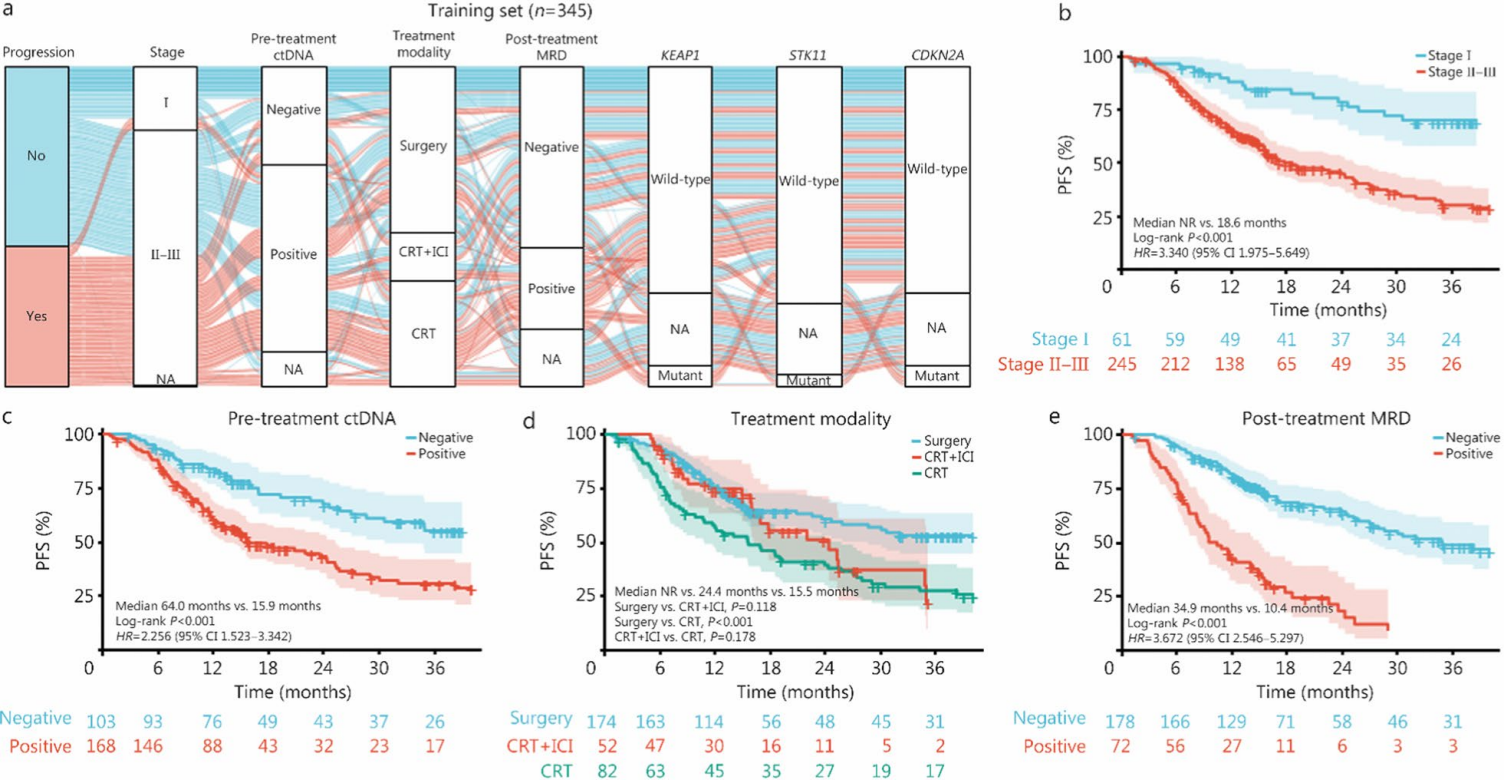
Predictive effects of identified multi-modality features in the training set (*n* = 345). **a** Sankey plot depicting identified variables in the training set (*n* = 345). **b** Kaplan–Meier curves of progression-free survival (PFS) stratified by stage in the training set (*n* = 306). Thirty-nine patients were excluded from analysis: 37 due to missing time-to-event data and 2 due to lack of stage information. **c** Kaplan–Meier curves of PFS stratified by pre-treatment ctDNA status (*n* = 271), with 37 patients excluded from analysis due to missing time-to-event data and 37 due to unavailable ctDNA testing results. **d** Kaplan–Meier curves of PFS stratified by treatment modalities (*n* = 308), with 37 patients excluded from analysis due to missing time-to-event data. **e** Kaplan–Meier curves of PFS stratified by post-treatment MRD (*n* = 250), with 37 patients excluded from analysis due to missing time-to-event data and 58 due to unavailable MRD. ctDNA circulating tumor DNA, MRD minimal residual disease, NA not available, CRT chemoradiotherapy, ICI immune checkpoint inhibitor, NR not reached, *HR* hazard ratio, CI confidence interval

On the basis of our above findings, several multi-modality features were applied to develop the PRIME model, including stage, pre-treatment ctDNA detection, treatment modality, post-treatment MRD, and mutations in *KEAP1*, *STK11*, and *CDKN2A*. In the training set (*n* = 345), most disease progression events occurred in patients characterized by stage II–III disease, baseline ctDNA positivity, CRT alone, and detectable MRD (Fig. 5a). The Sankey diagram also illustrated that, although some data were unavailable for several features (Additional file 1: Table S13), most of the missing values were MAR and no systematic redirection contradicting the MAR assumption was observed (Fig. 5a). For patients with stage II–III disease, PFS was significantly shorter than those with stage I disease (median 18.6 months vs. NR; *P* < 0.001; *HR* = 3.340, 95% CI 1.975–5.649; Fig. 5b). Moreover, patients with positive ctDNA at baseline had significantly poorer PFS than those with negative ctDNA (median 15.9 months vs. 64.0 months; *P* < 0.001; *HR* = 2.256, 95% CI 1.523–3.342; Fig. 5c). Resectable patients treated with surgery had significantly longer PFS than unresectable patients receiving CRT alone (median NR vs. 15.5 months; *P* < 0.001); however, PFS for patients who underwent surgery was numerically longer than PFS for unresectable patients who received CRT plus consolidation ICI (median NR vs. 24.4 months;* P* = 0.118; Fig. 5d). Patients with detectable MRD had significantly shorter PFS than those with undetectable MRD (median 10.4 months vs. 34.9 months; *P* < 0.001; *HR* = 3.672, 95% CI 2.546–5.297; Fig. 5e). Overall, each of the above factors could effectively predict the risk of progression when used individually. Thus, we integrated the aforementioned key features into a model that employs ML algorithms to enhance predictive accuracy.

### Superior prediction performance of the NN-based PRIME model

**Fig. 6:**
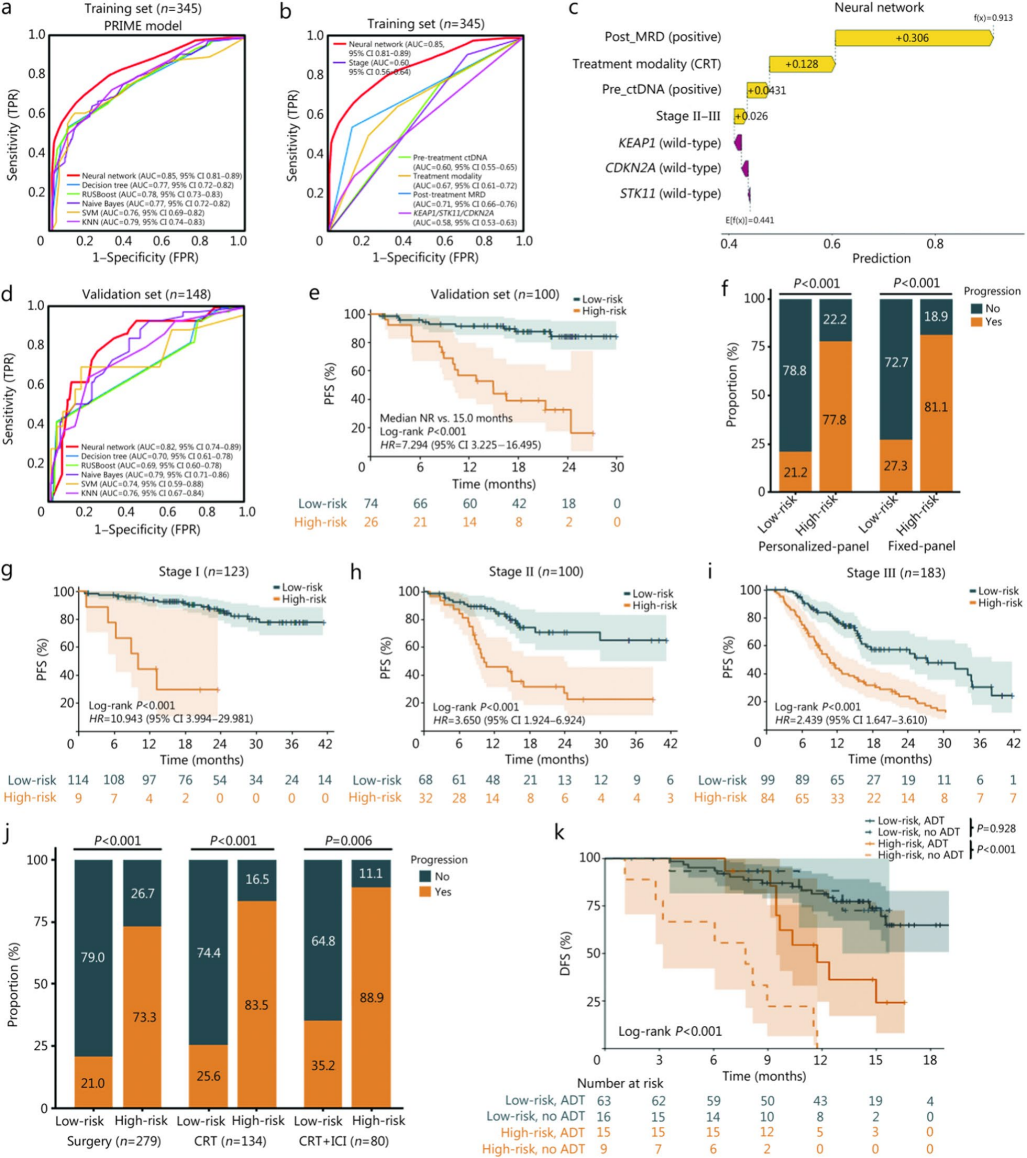
Neural network (NN)-based PRIME model construction, interpretation, and validation. **a** Receiver operating characteristic (ROC) curves of 6 different machine learning algorithms to develop the PRIME model in the training set (*n* = 345). **b** ROC curves comparing the NN-PRIME model to 5 individual features. **c** SHapley Additive exPlanations (SHAP) analysis of the NN-PRIME model. **d** ROC curves of 6 different machine learning algorithms to validate the PRIME model in the validation set (*n* = 148). **e** Kaplan–Meier curves of progression-free survival (PFS) stratified by progression risk predicted by NN-PRIME for patients in the validation set (*n* = 100). Forty-eight patients were excluded from analysis due to missing time-to-event data. **f** Stacked bar plots showing the proportion of progression (orange bar) in low-risk vs. high-risk patients using personalized-panel or fixed-panel ctDNA sequencing techniques. **g** Kaplan–Meier curves of PFS stratified by NN-PRIME for patients with stage I disease (*n* = 123), with 7 stage I patients excluded from analysis due to missing time-to-event data. Eighty-five patients without time-to-event data, and 2 without stage information, were excluded from analysis. **h** Kaplan–Meier curves of PFS stratified by NN-PRIME for patients with stage II disease (*n* = 100), with 11 stage II patients excluded from analysis due to missing time-to-event data. **i** Kaplan–Meier curves of PFS stratified by NN-PRIME for patients with stage III disease (*n* = 183), with 54 stage III patients excluded from analysis due to missing time-to-event data. **j** Stacked bar plots showing the proportion of disease progression (orange bar) in low-risk vs. high-risk patients identified by NN-PRIME, in patients receiving surgery, CRT alone, or CRT plus consolidation ICI therapy. **k** Kaplan–Meier curves of disease-free survival (DFS) stratified by NN-PRIME and adjuvant therapy (ADT) in the NCC-2 cohort (*n* = 103). TPR true positive rate, AUC area under the curve, RUSBoost random under-sampling boosting, SVM support vector machine, KNN K-nearest neighbors, FPR false positive rate, MRD minimal residual disease, ctDNA circulating tumor DNA, Post post-treatment, CRT chemoradiotherapy, Pre pre-treatment, NN neural network, NR not reached, *HR* hazard ratio, CI confidence interval, ICI immune checkpoint inhibitor, ADT adjuvant therapy, PRIME Progression Risk prediction by Interpretable Machine learning on ctDNA-MRD, Mutations, and clinical-therapeutic features

We first developed a logistic regression model by simply combining the efficient features together to predict the risk of progression, and the AUC for this combined model was 0.79 (95% CI 0.74–0.85; Additional file 2: Fig. S6). To further increase the predictive effects, we constructed ML-based models using 6 different ML algorithms. The NN-based PRIME model demonstrated optimal prediction performance, with an AUC of 0.85 (95% CI 0.81–0.89) in the training set, compared with decision tree (AUC = 0.77, 95% CI 0.72–0.82; DeLong’s test *P* = 0.0001), RUSBoost (AUC = 0.78, 95% CI 0.73–0.83; DeLong’s test *P* = 0.0002), naive Bayes (AUC = 0.77, 95% CI 0.72–0.82; DeLong’s test *P* = 0.0006), SVM (AUC = 0.76, 95% CI 0.69–0.82; DeLong’s test *P* < 0.0001), and KNN (AUC = 0.79, 95% CI 0.74–0.83; DeLong’s test *P* = 0.0093; Fig. 6a). F1-score, accuracy, precision, and recall for each model are summarized in Additional file 1: Table S14. The NN-PRIME model (AUC = 0.85, 95% CI 0.81–0.89) was significantly superior to individual predictors, including clinical stage (AUC = 0.60, 95% CI 0.56–0.64; DeLong’s test *P* < 0.0001), pre-treatment ctDNA (AUC = 0.60, 95% CI 0.55–0.65; DeLong’s test *P* < 0.0001), treatment modality (AUC = 0.67, 95% CI 0.61–0.72; DeLong’s test *P* < 0.0001), post-treatment MRD (AUC = 0.71, 95% CI 0.66–0.76; DeLong’s test *P* < 0.0001), and mutations in *KEAP1*, *STK11,* and *CDKN2A* (AUC = 0.58, 95% CI 0.53–0.63; DeLong’s test *P* < 0.0001; Fig. 6b). Given the positive correlation between *KEAP1* and *STK11*, as well as the adverse prognostic impact of *KEAP1*/*STK11* co-mutations, we tested *KEAP1*/*STK11* as a composite variable. However, merging *KEAP1* and *STK11* into a single feature did not improve model performance compared with treating *KEAP1*, *STK11,* and *CDKN2A* as 3 separate features (AUC 0.85, 95% CI 0.81–0.89 vs. 0.85, 95% CI 0.81–0.89 in the training set; AUC 0.79, 95% CI 0.71–0.87 vs. 0.82, 95% CI 0.74–0.89 in the validation set; Additional file 2: Fig. S7). Ultimately, the NN-PRIME model retained the following 7 key predictors: stage, pre-treatment ctDNA, treatment modality, post-treatment MRD, and mutations in *KEAP1*, *STK11*, and *CDKN2A*.

To further interpret the model, all contributing features were ranked with SHAP analysis. MRD, treatment modality, and baseline ctDNA status emerged as the top 3 contributors, both in terms of SHAP values and overall contribution (Additional file 2: Fig. S8). Specifically, the average predicted value across all samples was 0.441, and post-treatment MRD contributed positively to the NN-PRIME prediction (+0.306), suggesting an increased likelihood of predicting the positive class (Fig. 6c). CRT alone also contributed positively (+0.128). Detectable baseline ctDNA and stage II–III disease modestly increased the predicted probability (+0.043 and +0.026, respectively). In contrast, wild-type *KEAP1*, *STK11*, and *CDKN2A* mutations made negative contributions to the model output of progression (Fig. 6c).

Moreover, we evaluated ML-based models in the validation set, where NN-PRIME maintained good performance with an AUC of 0.82 (95% CI 0.74–0.89), and the AUCs were 0.70 (95% CI 0.61–0.78) for decision tree, 0.69 (95% CI 0.60–0.78) for RUSBoost, 0.79 (95% CI 0.71–0.86) for naive Bayes, 0.74 (95% CI 0.59–0.88) for SVM, and 0.76 (95% CI 0.67–0.84) for KNN (Fig. 6d). Hence, according to the predicted results of NN-PRIME, patients were classified into low- or high-risk groups of disease progression. In the validation set, high-risk patients had significantly poorer PFS than low-risk patients (median 15.0 months vs. NR; *P* < 0.001; *HR* = 7.294, 95% CI 3.225–16.495; Fig. 6e). For all patients or patients in the training set, the PFS of the high-risk group was significantly shorter than that of the low-risk group (*P* < 0.001; Additional file 2: Fig. S9).

### Robustness and applicability of NN-PRIME across diverse clinical scenarios

Next, we tested the generalizability and reliability of the NN-PRIME model. Sensitivity analyses based on complete-case analysis yielded virtually identical AUCs (0.85 vs. 0.83 in the training set; 0.82 vs. 0.80 in the validation set), confirming good model robustness (Additional file 2: Fig. S10). NN-PRIME effectively identified the risk of treatment failure (Fig. 6f) and stratified PFS (*P* < 0.001; Additional file 2: Fig. S11) irrespective of whether personalized-panel (*n* = 200) or fixed-panel (*n* = 293) ctDNA sequencing techniques were used. We also estimated ctDNA panel-specific random intercepts using GLMM, and no significant between-panel heterogeneity was observed (variance = 0, likelihood-ratio test *P* > 0.99; Additional file 1: Table S15). Subgroup analyses further illustrated that, after cross-platform harmonization, NN-PRIME retained consistent performance regardless of sequencing platform, ctDNA detection technique, or patient cohort (Additional file 2: Fig. S12), highlighting that technical variability did not compromise predictive accuracy.

High-risk patients identified by NN-PRIME had significantly worse PFS, irrespective of whether patients were at stage I (*P* < 0.001; *HR* = 10.943, 95% CI 3.994–29.981; Fig. 6g), stage II (*P* < 0.001; *HR* = 3.650, 95% CI 1.924–6.924; Fig. 6h), or stage III disease (*P* < 0.001; *HR* = 2.439, 95% CI 1.647–3.610; Fig. 6i). In terms of treatment regimens, for patients receiving surgery (*n* = 279; progression occurred in 21.0% of low-risk patients vs. 73.3% of high-risk patients; *P* < 0.001), CRT alone (*n* = 134; progression occurred in 25.6% of low-risk patients vs. 83.5% of high-risk patients; *P* < 0.001), or CRT plus consolidation ICI (*n* = 80; progression occurred in 35.2% of low-risk patients vs. 88.9% of high-risk patients; *P* = 0.006), NN-PRIME exhibited consistency in predicting treatment failure risk (Fig. 6j) and survival outcomes (Additional file 2: Fig. S13). In patients with resectable or unresectable stage II–III NSCLC, based on the treatment administered, the high-risk subgroup had a significantly greater risk of disease progression (*P* < 0.001; Additional file 2**:** Fig. S14). Subgroup analyses indicated that across all the clinical subgroups, high-risk patients identified by PRIME had a significantly greater risk of disease progression (*P* < 0.05; Additional file 2: Fig. S15). Notably, for patients with resectable NSCLC in the NCC-2 cohort, we observed that high-risk patients could significantly benefit more from adjuvant therapy (ADT) after radical surgery (*P* < 0.001), whereas there was no significant difference between low-risk patients with or without ADT (*P* = 0.928; Fig. 6k). In summary, NN-PRIME manifested good robustness and applicability across diverse clinical settings. The high-risk patient subgroup, which faced a greater risk of treatment failure, was poised to benefit significantly from intensified ADT strategies.

## Discussion

With increasing evidence from translational studies attesting to the predictive benefit of liquid biopsies, their use is being promoted and applied in real-world clinical practice [42–45]. Although ctDNA-MRD has achieved success in identifying disease relapse early, the predictive power of single liquid biopsy biomarkers is limited, with inconsistent predictive ability in various clinical scenarios [9, 10]. To date, large-scale data derived from different sequencing platforms that use blood samples from diverse populations, to test the generalizability of ctDNA-based predictions are lacking [46]. Using a global dataset of liquid biopsy and clinical-genomic variables from 493 patients with localized NSCLC, we developed and externally validated the NN-PRIME model. NN-PRIME achieved AUCs of 0.85 (95% CI 0.81–0.89) and 0.82 (95% CI 0.74–0.89) in the training and validation sets, respectively, outperforming single MRD testing and other machine-learning algorithms (all DeLong’s test *P* < 0.05). These data suggest that integrating ctDNA MRD with blood-based mutations and clinical features could refine post-treatment risk stratification and facilitate early, personalized clinical decision-making.

Compared with most traditional ML prediction studies, NN-PRIME was built on clinically relevant variables. The genomic components were further corroborated by external tissue WES/WGS analyses and transcriptomic profiling, and the contribution of each feature was quantified by SHAP analysis. These steps may collectively enhance the interpretability and predictive performance of NN-PRIME for therapeutic outcomes. Instead of using all factors shared among participants or simply performing feature ranking calculations, such as random forest methods, to include variables in the ML model development, we selected predictors essentially impacting clinical outcomes to construct PRIME. This could be one of the key reasons why the PRIME model has good performance and excellent generalizability across different patient populations and treatment regimens. Previous publications have also compared the performance of various ML algorithms in predicting outcomes [47–49]. Chang et al. [19] introduced a six-feature logistic LASSO regression model to predict response to immunotherapy (LORIS) using pan-cancer data. Six features were utilized, including age, tumor mutational burden (TMB), blood albumin level, neutrophil–lymphocyte ratio [19]. However, the authors identified these 6 features from 8 variables shared by most subjects using feature importance analysis [19], which may lead to insufficient interpretability of the included factors. Specifically, the predictive impact of age and blood albumin on the objective response to immunotherapy is uncertain. This could also explain why LORIS had AUC values of 0.66–0.77 in patients with NSCLC [19], while NN-PRIME could achieve an AUC of 0.85.

In addition to the interpretable features and enhanced predictive performance of PRIME, another obvious advantage is the readily accessible predictors, which provide great convenience for the real-world application of this model. Especially for patients with unresectable localized NSCLC, obtaining adequate tumor tissue samples for genetic profiling is difficult [50]. Our findings suggest that genetic mutations, including *KEAP1*, *STK11*, and *CDKN2A*, identified in peripheral blood could effectively predict outcomes as well. On this basis, combining blood-based genomic features with ctDNA-MRD and clinical characteristics can achieve more robust prediction of outcomes, providing guidance for risk-adaptive ADT. Zhao et al. [49] constructed a decision tree model (DT10) to predict immunotherapy efficacy in patients with metastatic NSCLC using tissue-based mutation, histology, TMB, etc., and the AUC of this tumor tissue-based model ranged between 0.79 and 0.83 [49], which is comparable to that of our blood-based PRIME model (AUC 0.82–0.85), suggesting that the predictive model based on liquid biopsy has similar reliability to the tissue-derived predictions. Given that Zhao et al. [49] also identified the pivotal prognostic effect of *KEAP1/STK11* mutations, this could further confirm our findings from the dimension of tumor tissue.

We observed that *STK11*, *KEAP1*, and *CDKN2A* could increase the risk of disease recurrence. RNA-seq analyses further revealed significantly decreased infiltration of eosinophils, mast cells and T-helper 17 cells, together with downregulated macrophage differentiation, mast-cell activation and B-cell-mediated immunity, in *STK11*-, *KEAP1*-, and *CDKN2A*-mutated tumors, indicating the suppression of humoral immune function collectively driven by these cells and pathways. Although previous studies have shown that tumor tissue-based *STK11*, *KEAP1*, and *CDKN2A/B* mutations are adverse prognostic factors [51, 52], we further confirm that ctDNA-based *STK11*, *KEAP1*, and *CDKN2A* can predict unfavorable outcomes in patients with non-metastatic NSCLC. Prior research has suggested that tumors with *STK11*, *KEAP1*, or *CDKN2A* mutations are characterized by attenuation of T cell-mediated adaptive immunity or innate immunity [53, 54]. Our findings further elucidate that the downregulation of innate humoral immune responses may be a more important reason for their adverse prognostic impacts. We also observed that modeling *KEAP1/STK11* co-mutation as a single feature did not improve prediction over treating the two mutations separately. This reflects that merging *KEAP1* and *STK11* into one variable may prevent the model from capturing biologically relevant nuances. For instance, *KEAP1* primarily affects the Nrf2-antioxidant signaling pathway, *STK11* loss impairs cell polarity and energy metabolism, and *CDKN2A* mainly exerts influence through cell-cycle regulation [55–57]. Compressing these mutations into one composite feature risks obscuring these nuanced, pathway-specific impacts and their complex interplay with clinical factors such as disease stage and ctDNA status. Retaining *KEAP1*, *STK11*, and *CDKN2A* as separate inputs allows the model to exploit their individual prognostic value and thereby enhances predictive accuracy.

It is noteworthy that PRIME can successfully identify the high-risk subgroup in early-stage NSCLC patients, particularly in stage I (*HR* = 10.943, 95% CI 3.994–29.981) and stage II (*HR* = 3.650, 95% CI 1.924–6.924) patients. In the NCC-2 cohort, PRIME-defined high-risk patients derived marked benefit from post-surgical ADT (*P* < 0.001), whereas no significant survival difference was observed between low-risk patients with or without ADT (*P* = 0.928). According to CheckMate-816 [58], IMpower010 [59], and KEYNOTE-091 [60], perioperative immunotherapy brings therapeutic benefits to patients with resectable stage IB–IIIA NSCLC. However, not all early-stage patients could benefit from perioperative immunotherapy. In the CheckMate-816 trial, there was no significant difference in event-free survival between stage IB–II patients receiving neoadjuvant chemotherapy alone and those receiving chemoimmunotherapy [58]. Therefore, early and precise identification of patient subgroups with poorer prognoses and higher risks of treatment failure is vital for patients with early-stage NSCLC to guide personalized and risk-adaptive perioperative immunotherapy [61, 62].

The more pronounced risk stratification effect of NN-PRIME in early-stage patients may be attributed to biological differences across stages. Early-stage tumors typically exhibit lower genomic complexity and fewer subclonal evolutions, allowing liquid biopsy-based genotyping to more reliably capture dominant aggressive clones [63, 64]. In contrast, locally advanced stage III tumors often harbor greater intratumoral heterogeneity, which may dilute the prognostic signal of blood-derived mutations at baseline [65, 66]. Besides, the predictive role of ctDNA-MRD status is more corroborative in early-stage NSCLC patients who undergo surgical resection, while the accuracy of post-CRT MRD in predicting outcomes may be confounded by the impact of consolidation immunotherapy [44]. Furthermore, we revealed that NN-PRIME achieved superior risk stratification in patients undergoing surgery or CRT alone (*P* < 0.001), compared to those receiving CRT plus consolidation ICIs (*P* = 0.006). This differential performance may stem from PRIME-identified high-risk patients having inherently poorer outcomes and thus potentially deriving greater benefits from consolidation immunotherapy after CRT. This also indirectly reflects PRIME’s potential to guide personalized and risk-adaptive treatment decisions, particularly as we demonstrated that high-risk patients benefited substantially from intensified ADT after surgery, while low-risk patients showed no significant benefit. In the future, the PRIME model will be verified in a prospective, multicenter, phase II trial (ClinicalTrials.gov identifier: NCT06998719). This study will enroll localized NSCLC patients receiving neoadjuvant immunotherapy followed by either surgery plus adjuvant immunotherapy, or definitive CRT plus consolidation immunotherapy, aiming to further confirm the prognostic performance of the model and to evaluate its utility for predicting benefit from perioperative immunotherapy.

This study had several limitations. First, research subjects had a certain degree of heterogeneity, comprising patients with stage I–III NSCLC, resectable or unresectable disease, and those treated with curative-intent surgery or radiotherapy, which may introduce potential bias. Nevertheless, NN-PRIME maintained robust prediction across all examined subgroups. Second, ctDNA profiling was performed using different techniques, including fixed-panel and personalized-panel. This is consistent with the situation in which patients receive ctDNA testing in the real world, and PRIME effectively predicts the risk of disease progression in patient subgroups using different ctDNA sequencing platforms, providing a reference for real-world clinical practice. Third, owing to limited data resources, we analyzed disease progression risk and PFS, but not OS. However, there is high-level evidence indicating that DFS and PFS are valid surrogate endpoints for OS in patients with early-stage and localized NSCLC [67]. In addition, there is some missing data in certain analyses, which should be taken into account when generalizing the findings. Lastly, the mechanisms underlying the adverse prognostic impact of *STK11*, *KEAP1*, and *CDKN2A* mutations, currently inferred from tumor RNA-seq data and bioinformatic analyses, remain to be experimentally validated in future in vivo and in vitro fundamental research.

## Conclusions

In conclusion, we developed PRIME, an interpretable AI-driven model trained on global datasets. After integrating liquid biopsy biomarkers, genomic mutations, with other clinical informative features, PRIME enabled early identification of progression risk in stage I–III NSCLC patients treated with curative intent. The NN-PRIME model demonstrated superior predictive performance compared to single ctDNA-MRD assays and other ML models (all DeLong’s test *P* < 0.05), with robust applicability and practicality across diverse clinical scenarios. In addition, patients identified as high-risk by NN-PRIME showed significantly worse prognoses but could potentially derive greater benefits from ADT, highlighting the potential of NN-PRIME to facilitate risk-stratified clinical decision-making. Future investigation is warranted to prospectively explore the incorporation of PRIME into routine clinical practice to improve patient outcomes and advance therapeutic personalization.

## Supplementary Information

**Additional file 1**. **Table S1** Baseline characteristics of patients from all cohorts (*n* = 493). **Table S2 **Clinical, treatment and ctDNA data for patients in the NCC-1 cohort (*n* = 105). **Table S3** Clinical, treatment and ctDNA data for patients in the NCC-2 cohort (*n* = 103). **Table S4** Clinical, treatment and ctDNA data for patients in the Stanford cohort (*n* = 37). **Table S5** Clinical, treatment and ctDNA data patients in the LUCID cohort (*n* = 100). **Table S6** Clinical, treatment and ctDNA data for patients in the TRACERx cohort (*n* = 100). **Table S7** Clinical, treatment, and ctDNA data for patients in the Moding et al. cohort (*n* = 48). **Table S8** Mutational data of patients in the NCC-1 cohort (*n* = 105). **Table S9** Mutational data of patients in the NCC-2 cohort (*n* = 103). **Table S10** Comparison of baseline characteristics of patients in the training set and validation set. **Table S11** The VIF for each feature in the logistic regression analysis. **Table S12** Baseline information and variants data for patients in the TCGA WES/WGS validation cohort (*n* = 430). **Table S13** Missing data summary in the training set (*n* = 345). **Table S14** F1-score, accuracy, precision, and recall for each model. **Table S15** Generalized linear mixedeffects model of disease progression across different ctDNA detection techniques.

**Additional file 2**. **Fig. S1** Survival outcomes. **Fig. S2** RNA-sequencing (RNA-seq) analysis of *KEAP1, STK11*, and *CDKN2A* mutations in the Cancer Genome Atlas (TCGA) database. **Fig. S3** Prognostic effects of *KEAP1/STK11* mutations in the overall patient cohort. **Fig. S4** Immune infiltration analysis of *KEAP1/STK11/CDKN2A* mutations. **Fig. S5** Gene set enrichment analysis (GSEA) ridge plots showing the normalized enrichment score (NES), adjusted P-value, and false discovery rate (FDR) for significantly enriched gene sets. **Fig. S6** Receiver operating characteristic (ROC) curve for the combined model based on logistic regression. **Fig. S7** Comparison of model performance with composite vs. separate genomic features. **Fig. S8** SHapley Additive exPlanations (SHAP) for neural network (NN)-based PRIME model. **Fig. S9** Progression-free survival stratified by NN-PRIME in all patients (**a**) and in the training set (**b**). **Fig. S10** Sensitivity analyses of NN-PRIME model robustness. **Fig. S11** Progression-free survival stratified by NN-PRIME in patients receiving personalized-panel (tumor-informed) ctDNA testing (**a**) and in patients receiving fixed-panel (tumor-naïve) ctDNA testing (**b**). **Fig. S12** Subgroup analyses of NN-PRIME model performance in different ctDNA sequencing platforms, detection techniques, and patient cohorts. **Fig. S13** Kaplan-Meier curves of survival outcomes in patients with different treatment regimens. **Fig. S14** Proportion of disease progression in low-risk vs. high-risk patients stratified by NN-PRIME in resectable or unresectable stage II–III NSCLC patients. **Fig. S15** Forest plot of logistic regression indicating high-risk patients identified by NN-PRIME correlated with poorer outcomes across various clinical subgroups.

## Data Availability

The datasets supporting the main conclusions of this article are included within the article and supplementary materials. The raw sequencing data of NCC-1 and NCC-2 cohorts are deposited in the Genome Sequence Archive (GSA) for Human in National Genomics Data Center, under the accession numbers of HRA011737 and HRA001346. Detailed anonymous survival data for each patient will be available from the corresponding authors on reasonable request.

## Abbreviations

ADT: Adjuvant therapy
AI: Artificial intelligence
AUC: Area under the curve
CDKN2A: Cyclin-dependent kinase inhibitor 2a
CI: Confidence interval
CRT: Chemoradiotherapy
ctDNA: Circulating tumor DNA
DFS: Disease-free survival
EGFR: Epidermal growth factor receptor
ERBB2: Erb-B2 receptor tyrosine kinase 2
FDR: False discovery rate
GLMM: Generalized linear mixed-effects model
GO/KEGG: Gene Ontology/Kyoto Encyclopedia of Genes and Genomes
GSEA: Gene set enrichment analysis
*HR*: Hazard ratio
ICIs: Immune checkpoint inhibitors
KEAP1: Kelch-like ECH-associated protein 1
KNN: K-nearest neighbors
KRAS: Kirsten rat sarcoma viral oncogene homolog
LUSC: Lung squamous cell carcinoma
LUAD: Lung adenocarcinoma
MAR: Missing at random
MCAR: Missing completely at random
MET: Mesenchymal-epithelial transition factor
MNAR: Missing not at random
MRD: Minimal residual disease
ML: Machine learning
NES: Normalized enrichment score
NN: Neural network
NSCLC: Non-small cell lung cancer
*OR*: Odds ratio
OS: Overall survival
PFS: Progression-free survival
PIK3CA: Phosphatidylinositol-4,5-bisphosphate 3-kinase catalytic subunit alpha
PTEN: Phosphatase and tensin homolog
RECIST: Response Evaluation Criteria in Solid Tumors
RNA-seq: RNA-sequencing
ROC: Receiver operating characteristic
RUSBoost: Random under-sampling boosting
SHAP: SHapley Additive exPlanations
SMARCA4: SWI/SNF-related, matrix-associated, actin-dependent regulator of chromatin, subfamily A, member 4
SNVs: Single-nucleotide variants
STK11: Serine/threonine kinase 11
SVM: Support vector machine
TCGA: The Cancer Genome Atlas
TIME: Tumor immune microenvironment
TMB: Tumor mutational burden
TP53: Tumor protein p53
VIF: Variance inflation factor
WES: Whole-exome sequencing
WGS: Whole-genome sequencing

## References

[1] Siegel RL, Miller KD, Wagle NS, Jemal A. Cancer statistics, 2023. CA Cancer J Clin. 2023;73(1):17–48. 36633525 10.3322/caac.21763

[2] Thuya WL, Peyper JM, Myen TT, Anuar ND, Anwar A, Gudimella R, et al. Exosome autoantibody biomarkers for detection of lung cancer. Mil Med Res. 2024;11(1):72. 39558443 10.1186/s40779-024-00575-y PMC11571996

[3] Postmus PE, Kerr KM, Oudkerk M, Senan S, Waller DA, Vansteenkiste J, et al. Early and locally advanced non-small-cell lung cancer (NSCLC): esmo clinical practice guidelines for diagnosis, treatment and follow-up. Ann Oncol. 2017;28(suppl_4):iv1–21. 28881918 10.1093/annonc/mdx222

[4] Sujit SJ, Aminu M, Karpinets TV, Chen P, Saad MB, Salehjahromi M, et al. Enhancing NSCLC recurrence prediction with PET/CT habitat imaging, ctDNA, and integrative radiogenomics-blood insights. Nat Commun. 2024;15(1):3152. 38605064 10.1038/s41467-024-47512-0 PMC11009351

[5] Hung JJ, Hsu WH, Hsieh CC, Huang BS, Huang MH, Liu JS, et al. Post-recurrence survival in completely resected stage I non-small cell lung cancer with local recurrence. Thorax. 2009;64(3):192–6. 19252018 10.1136/thx.2007.094912

[6] Wilson BE, Wright K, Koven R, Booth CM. Surveillance imaging after curative-intent treatment for cancer: benefits, harms, and evidence. J Clin Oncol. 2024;42(19):2245–9. 38805665 10.1200/JCO.23.02475

[7] Larici AR, del Ciello A, Maggi F, Santoro SI, Meduri B, Valentini V, et al. Lung abnormalities at multimodality imaging after radiation therapy for non-small cell lung cancer. Radiographics. 2011;31(3):771–89. 21571656 10.1148/rg.313105096

[8] Lee K, Le T, Hau E, Hanna GG, Gee H, Vinod S, et al. A systematic review into the radiologic features predicting local recurrence after stereotactic ablative body radiotherapy (SABR) in patients with non-small cell lung cancer (NSCLC). Int J Radiat Oncol Biol Phys. 2022;113(1):40–59. 34879247 10.1016/j.ijrobp.2021.11.027

[9] Abbosh C, Birkbak NJ, Swanton C. Early stage NSCLC - challenges to implementing ctDNA-based screening and MRD detection. Nat Rev Clin Oncol. 2018;15(9):577–86. 29968853 10.1038/s41571-018-0058-3

[10] Pellini B, Chaudhuri AA. Circulating tumor DNA minimal residual disease detection of non-small-cell lung cancer treated with curative intent. J Clin Oncol. 2022;40(6):567–75. 34985936 10.1200/JCO.21.01929 PMC8853615

[11] Kilgour E, Rothwell DG, Brady G, Dive C. Liquid biopsy-based biomarkers of treatment response and resistance. Cancer Cell. 2020;37(4):485–95. 32289272 10.1016/j.ccell.2020.03.012

[12] Tie J, Wang Y, Tomasetti C, Li L, Springer S, Kinde I, et al. Circulating tumor DNA analysis detects minimal residual disease and predicts recurrence in patients with stage II colon cancer. Sci Transl Med. 2016;8(346):346ra92. 27384348 10.1126/scitranslmed.aaf6219 PMC5346159

[13] Garcia-Murillas I, Schiavon G, Weigelt B, Ng C, Hrebien S, Cutts RJ, et al. Mutation tracking in circulating tumor DNA predicts relapse in early breast cancer. Sci Transl Med. 2015;7(302):302ra133. 26311728 10.1126/scitranslmed.aab0021

[14] Chabon JJ, Hamilton EG, Kurtz DM, Esfahani MS, Moding EJ, Stehr H, et al. Integrating genomic features for non-invasive early lung cancer detection. Nature. 2020;580(7802):245–51. 32269342 10.1038/s41586-020-2140-0 PMC8230734

[15] Shitara K, Muro K, Watanabe J, Yamazaki K, Ohori H, Shiozawa M, et al. Baseline ctDNA gene alterations as a biomarker of survival after panitumumab and chemotherapy in metastatic colorectal cancer. Nat Med. 2024;30(3):730–9. 38347302 10.1038/s41591-023-02791-w PMC10957476

[16] Kurtz DM, Esfahani MS, Scherer F, Soo J, Jin MC, Liu CL, et al. Dynamic risk profiling using serial tumor biomarkers for personalized outcome prediction. Cell. 2019;178(3):699-713.e19. 31280963 10.1016/j.cell.2019.06.011 PMC7380118

[17] Carrasco-Zanini J, Pietzner M, Davitte J, Surendran P, Croteau-Chonka DC, Robins C, et al. Proteomic signatures improve risk prediction for common and rare diseases. Nat Med. 2024;30(9):2489–98. 39039249 10.1038/s41591-024-03142-z PMC11405273

[18] Chen Y, Wang B, Zhao Y, Shao X, Wang M, Ma F, et al. Metabolomic machine learning predictor for diagnosis and prognosis of gastric cancer. Nat Commun. 2024;15(1):1657. 38395893 10.1038/s41467-024-46043-y PMC10891053

[19] Chang TG, Cao Y, Sfreddo HJ, Dhruba SR, Lee SH, Valero C, et al. LORIS robustly predicts patient outcomes with immune checkpoint blockade therapy using common clinical, pathologic and genomic features. Nat Cancer. 2024;5(8):1158–75. 38831056 10.1038/s43018-024-00772-7 PMC11962634

[20] Yuan Y, Zhang X, Wang Y, Li H, Qi Z, Du Z, et al. Multimodal data integration using deep learning predicts overall survival of patients with glioma. VIEW. 2024;5(5):20240001.

[21] Le NQK, Li W, Cao Y. Sequence-based prediction model of protein crystallization propensity using machine learning and two-level feature selection. Brief Bioinform. 2023;24(5):bbad319. 37649385 10.1093/bib/bbad319

[22] Kha QH, Le VH, Hung TNK, Le NQK. Development and validation of an efficient MRI radiomics signature for improving the predictive performance of 1p/19q co-deletion in lower-grade gliomas. Cancers (Basel). 2021;13(21):5398. 34771562 10.3390/cancers13215398 PMC8582370

[23] Greener JG, Kandathil SM, Moffat L, Jones DT. A guide to machine learning for biologists. Nat Rev Mol Cell Biol. 2022;23(1):40–55. 34518686 10.1038/s41580-021-00407-0

[24] Chouaïd C, Gendarme S, Auliac JB. Artificial intelligence to finally enable precision medicine for the management of resected non-small-cell lung cancer. Ann Oncol. 2023;34(7):565–6. 37182802 10.1016/j.annonc.2023.05.001

[25] Swanson K, Wu E, Zhang A, Alizadeh AA, Zou J. From patterns to patients: advances in clinical machine learning for cancer diagnosis, prognosis, and treatment. Cell. 2023;186(8):1772–91. 36905928 10.1016/j.cell.2023.01.035

[26] Azodi CB, Tang J, Shiu SH. Opening the black box: interpretable machine learning for geneticists. Trends Genet. 2020;36(6):442–55. 32396837 10.1016/j.tig.2020.03.005

[27] Li LS, Yang L, Zhuang L, Ye ZY, Zhao WG, Gong WP. From immunology to artificial intelligence: revolutionizing latent tuberculosis infection diagnosis with machine learning. Mil Med Res. 2023;10(1):58. 38017571 10.1186/s40779-023-00490-8 PMC10685516

[28] Gale D, Heider K, Ruiz-Valdepenas A, Hackinger S, Perry M, Marsico G, et al. Residual ctDNA after treatment predicts early relapse in patients with early-stage non-small cell lung cancer. Ann Oncol. 2022;33(5):500–10. 35306155 10.1016/j.annonc.2022.02.007 PMC9067454

[29] Abbosh C, Birkbak NJ, Wilson GA, Jamal-Hanjani M, Constantin T, Salari R, et al. Phylogenetic ctDNA analysis depicts early-stage lung cancer evolution. Nature. 2017;545(7655):446–51. 28445469 10.1038/nature22364 PMC5812436

[30] Jamal-Hanjani M, Wilson GA, McGranahan N, Birkbak NJ, Watkins TBK, Veeriah S, et al. Tracking the evolution of non-small-cell lung cancer. N Engl J Med. 2017;376(22):2109–21. 28445112 10.1056/NEJMoa1616288

[31] Chaudhuri AA, Chabon JJ, Lovejoy AF, Newman AM, Stehr H, Azad TD, et al. Early detection of molecular residual disease in localized lung cancer by circulating tumor DNA profiling. Cancer Discov. 2017;7(12):1394–403. 28899864 10.1158/2159-8290.CD-17-0716 PMC5895851

[32] Moding EJ, Liu Y, Nabet BY, Chabon JJ, Chaudhuri AA, Hui AB, et al. Circulating tumor DNA dynamics predict benefit from consolidation immunotherapy in locally advanced non-small cell lung cancer. Nat Cancer. 2020;1(2):176–83. 34505064 10.1038/s43018-019-0011-0 PMC8425388

[33] Eisenhauer EA, Therasse P, Bogaerts J, Schwartz LH, Sargent D, Ford R, et al. New response evaluation criteria in solid tumours: revised RECIST guideline (version 1.1). Eur J Cancer. 2009;45(2):228–47. 19097774 10.1016/j.ejca.2008.10.026

[34] Walia A, Tuia J, Prasad V. Progression-free survival, disease-free survival and other composite end points in oncology: improved reporting is needed. Nat Rev Clin Oncol. 2023;20(12):885–95. 37828154 10.1038/s41571-023-00823-5

[35] DeLong ER, DeLong DM, Clarke-Pearson DL. Comparing the areas under two or more correlated receiver operating characteristic curves: a nonparametric approach. Biometrics. 1988;44(3):837–45. 3203132

[36] Niu L, Thiele M, Geyer PE, Rasmussen DN, Webel HE, Santos A, et al. Noninvasive proteomic biomarkers for alcohol-related liver disease. Nat Med. 2022;28(6):1277–87. 35654907 10.1038/s41591-022-01850-y PMC9205783

[37] Cancer Genome Atlas Research Network. Comprehensive genomic characterization of squamous cell lung cancers. Nature. 2012;489(7417):519–25. 22960745 10.1038/nature11404 PMC3466113

[38] Cancer Genome Atlas Research Network. Comprehensive molecular profiling of lung adenocarcinoma. Nature. 2014;511(7511):543–50. 25079552 10.1038/nature13385 PMC4231481

[39] Skoulidis F, Heymach JV. Co-occurring genomic alterations in non-small-cell lung cancer biology and therapy. Nat Rev Cancer. 2019;19(9):495–509. 31406302 10.1038/s41568-019-0179-8 PMC7043073

[40] Skoulidis F, Araujo HA, Do MT, Qian Y, Sun X, Galan-Cobo A, et al. CTLA4 blockade abrogates KEAP1/STK11-related resistance to PD-(L)1 inhibitors. Nature. 2024;635(8038):462–71. 39385035 10.1038/s41586-024-07943-7 PMC11560846

[41] Shaverdashvili K, Burns TF. Advances in the treatment of KRAS^G12C^ mutant non-small cell lung cancer. Cancer. 2025;131(Suppl 1):e35783. 40172157 10.1002/cncr.35783 PMC11963745

[42] Lv J, Xu LX, Li ZX, Lin L, Wu CF, Quan TQ, et al. Longitudinal on-treatment circulating tumor DNA as a biomarker for real-time dynamic risk monitoring in cancer patients: the EP-SEASON study. Cancer Cell. 2024;42(8):1401-14.e4. 39059389 10.1016/j.ccell.2024.07.001

[43] Qian DC, Ulrich BC, Peng G, Zhao H, Conneely KN, Miller AH, et al. Outcomes stratification of head and neck cancer using pre- and post-treatment DNA methylation from peripheral blood. Int J Radiat Oncol Biol Phys. 2023;115(5):1217–28. 36410685 10.1016/j.ijrobp.2022.11.009 PMC12272450

[44] Wang Y, Wang W, Zhang T, Yang Y, Wang J, Li C, et al. Dynamic bTMB combined with residual ctDNA improves survival prediction in locally advanced NSCLC patients with chemoradiotherapy and consolidation immunotherapy. J Natl Cancer Cent. 2024;4(2):177–87. 39282582 10.1016/j.jncc.2024.01.008 PMC11390629

[45] Wang Y, Zhang T, Yang Y, Wang J, Li C, Xu X, et al. Longitudinal circulating tumour DNA dynamics predict failure patterns and efficacy of consolidation immunotherapy after chemoradiotherapy in locally advanced non-small-cell lung cancer. Clin Transl Med. 2024;14(3):e1619. 38450838 10.1002/ctm2.1619 PMC10918705

[46] Ignatiadis M, Sledge GW, Jeffrey SS. Liquid biopsy enters the clinic - implementation issues and future challenges. Nat Rev Clin Oncol. 2021;18(5):297–312. 33473219 10.1038/s41571-020-00457-x

[47] Chowell D, Yoo SK, Valero C, Pastore A, Krishna C, Lee M, et al. Improved prediction of immune checkpoint blockade efficacy across multiple cancer types. Nat Biotechnol. 2022;40(4):499–506. 34725502 10.1038/s41587-021-01070-8 PMC9363980

[48] Charoentong P, Finotello F, Angelova M, Mayer C, Efremova M, Rieder D, et al. Pan-cancer immunogenomic analyses reveal genotype-immunophenotype relationships and predictors of response to checkpoint blockade. Cell Rep. 2017;18(1):248–62. 28052254 10.1016/j.celrep.2016.12.019

[49] Zhao J, Wang L, Zhou A, Wen S, Fang W, Zhang L, et al. Decision model for durable clinical benefit from front- or late-line immunotherapy alone or with chemotherapy in non-small cell lung cancer. Med. 2024;5(8):981-97.e4. 38781965 10.1016/j.medj.2024.04.011

[50] Crowley E, Di Nicolantonio F, Loupakis F, Bardelli A. Liquid biopsy: monitoring cancer-genetics in the blood. Nat Rev Clin Oncol. 2013;10(8):472–84. 23836314 10.1038/nrclinonc.2013.110

[51] Provencio-Pulla M, Pérez-Parente D, Olson S, Hasan H, Balea BC, Rodríguez-Abreu D, et al. Identification of non-actionable mutations with prognostic and predictive value in patients with advanced or metastatic non-small cell lung cancer. Clin Transl Oncol. 2024;26(6):1384–94. 38183584 10.1007/s12094-023-03362-8 PMC11108921

[52] Skoulidis F, Goldberg ME, Greenawalt DM, Hellmann MD, Awad MM, Gainor JF, et al. *STK11/LKB1* mutations and PD-1 inhibitor resistance in *KRAS*-mutant lung adenocarcinoma. Cancer Discov. 2018;8(7):822–35. 29773717 10.1158/2159-8290.CD-18-0099 PMC6030433

[53] Zhang C. The negative relationship between patients with NSCLC harbored *STK11/KEAP1* copy number variation and immune microenvironment infiltration. J Transl Med. 2021;19(1):259. 34127019 10.1186/s12967-021-02924-0 PMC8201872

[54] Marzio A, Kurz E, Sahni JM, Di Feo G, Puccini J, Jiang S, et al. EMSY inhibits homologous recombination repair and the interferon response, promoting lung cancer immune evasion. Cell. 2022;185(1):169-83.e19. 34963055 10.1016/j.cell.2021.12.005 PMC8751279

[55] Bollong MJ, Lee G, Coukos JS, Yun H, Zambaldo C, Chang JW, et al. A metabolite-derived protein modification integrates glycolysis with *KEAP1-NRF2* signalling. Nature. 2018;562(7728):600–4. 30323285 10.1038/s41586-018-0622-0 PMC6444936

[56] Granot Z, Swisa A, Magenheim J, Stolovich-Rain M, Fujimoto W, Manduchi E, et al. LKB1 regulates pancreatic beta cell size, polarity, and function. Cell Metab. 2009;10(4):296–308. 19808022 10.1016/j.cmet.2009.08.010 PMC2790403

[57] Zhao R, Choi BY, Lee MH, Bode AM, Dong Z. Implications of genetic and epigenetic alterations of *CDKN2A* (p16^INK4a^) in cancer. EBioMedicine. 2016;8:30–9. 27428416 10.1016/j.ebiom.2016.04.017 PMC4919535

[58] Forde PM, Spicer J, Lu S, Provencio M, Mitsudomi T, Awad MM, et al. Neoadjuvant nivolumab plus chemotherapy in resectable lung cancer. N Engl J Med. 2022;386(21):1973–85. 35403841 10.1056/NEJMoa2202170 PMC9844511

[59] Felip E, Altorki N, Zhou C, Csőszi T, Vynnychenko I, Goloborodko O, et al. Adjuvant atezolizumab after adjuvant chemotherapy in resected stage IB-IIIA non-small-cell lung cancer (IMpower010): a randomised, multicentre, open-label, phase 3 trial. Lancet. 2021;398(10308):1344–57. 34555333 10.1016/S0140-6736(21)02098-5

[60] O’Brien M, Paz-Ares L, Marreaud S, Dafni U, Oselin K, Havel L, et al. Pembrolizumab versus placebo as adjuvant therapy for completely resected stage IB-IIIA non-small-cell lung cancer (PEARLS/KEYNOTE-091): an interim analysis of a randomised, triple-blind, phase 3 trial. Lancet Oncol. 2022;23(10):1274–86. 36108662 10.1016/S1470-2045(22)00518-6

[61] Xiao M, Wang L, Tang Q, Yang Q, Yang X, Zhu G, et al. Postoperative tumor treatment strategies: from basic research to clinical therapy. VIEW. 2024;5(3):20230117.

[62] Tang J, Tian Y, Wang S, Liu Y, Chen M, Yang X, et al. Early postoperative plasma circulating tumour DNA for molecular residue disease detection and recurrence risk evaluation in surgical non-small cell lung cancer. Clin Transl Med. 2024;14(10):e70056. 39394592 10.1002/ctm2.70056 PMC11469951

[63] Abbosh C, Frankell AM, Harrison T, Kisistok J, Garnett A, Johnson L, et al. Tracking early lung cancer metastatic dissemination in TRACERx using ctDNA. Nature. 2023;616(7957):553–62. 37055640 10.1038/s41586-023-05776-4 PMC7614605

[64] Nicoś M, Krawczyk P. Genetic clonality as the hallmark driving evolution of non-small cell lung cancer. Cancers (Basel). 2022;14(7):1813. 35406585 10.3390/cancers14071813 PMC8998004

[65] Black JRM, McGranahan N. Genetic and non-genetic clonal diversity in cancer evolution. Nat Rev Cancer. 2021;21(6):379–92. 33727690 10.1038/s41568-021-00336-2

[66] McGranahan N, Swanton C. Clonal heterogeneity and tumor evolution: past, present, and the future. Cell. 2017;168(4):613–28. 28187284 10.1016/j.cell.2017.01.018

[67] Mauguen A, Pignon JP, Burdett S, Domerg C, Fisher D, Paulus R, et al. Surrogate endpoints for overall survival in chemotherapy and radiotherapy trials in operable and locally advanced lung cancer: a re-analysis of meta-analyses of individual patients’ data. Lancet Oncol. 2013;14(7):619–26. 23680111 10.1016/S1470-2045(13)70158-X PMC3732017

