# Supplementary material for: PRIME: an interpretable artificial intelligence model based on liquid biopsy improves prediction of progression risk in non-small cell lung cancer
Source: Mil Med Res. 2026 Jan 6;12:94. doi: 10.1186/s40779-025-00679-z (PMC12771999; doi:10.1186/s40779-025-00679-z)
Supplement: Supplementary file 2 — Additional file 2. Fig. S1 Survival outcomes. Fig. S2 RNA-sequencing (RNA-seq) analysis of KEAP1, STK11, and CDKN2A mutations in the Cancer Genome Atlas (TCGA) database. Fig. S3 Prognostic effects of KEAP1/STK11 mutations in the overall patient cohort. Fig. S4 Immune infiltration analysis of KEAP1/STK11/CDKN2A mutations. Fig. S5 Gene set enrichment analysis (GSEA) ridge plots showing the normalized enrichment score (NES), adjusted P-value, and false discovery rate (FDR) for significantly enriched gene sets. Fig. S6 Receiver operating characteristic (ROC) curve for the combined model based on logistic regression. Fig. S7 Comparison of model performance with composite vs. separate genomic features. Fig. S8 SHapley Additive exPlanations (SHAP) for neural network (NN)-based PRIME model. Fig. S9 Progression-free survival stratified by NN-PRIME in all patients (a) and in the training set (b). Fig. S10 Sensitivity analyses of NN-PRIME model robustness. Fig. S11 Progression-free survival stratified by NN-PRIME in patients receiving personalized-panel (tumor-informed) ctDNA testing (a) and in patients receiving fixed-panel (tumor-naïve) ctDNA testing (b). Fig. S12 Subgroup analyses of NN-PRIME model performance in different ctDNA sequencing platforms, detection techniques, and patient cohorts. Fig. S13 Kaplan-Meier curves of survival outcomes in patients with different treatment regimens. Fig. S14 Proportion of disease progression in low-risk vs. high-risk patients stratified by NN-PRIME in resectable or unresectable stage II–III NSCLC patients. Fig. S15 Forest plot of logistic regression indicating high-risk patients identified by NN-PRIME correlated with poorer outcomes across various clinical subgroups. [file 40779_2025_679_MOESM2_ESM.pdf]

Methods

NCC-1 cohort

Study population and clinical procedures

This cohort comprised 105 non-small cell lung cancer (NSCLC) patients treated at National Cancer Center (NCC) in Beijing, China between 2018 and 2022. Patients with unresectable stage I – III NSCLC receiving definitive chemoradiotherapy (CRT) alone or followed by consolidation immune checkpoint inhibitors (ICIs) were consecutively included in the analysis. The prescribed dose for radiotherapy ranged from 56 to 66 Gy, combined with platinum-based chemotherapy. Consolidation ICI therapy included programmed cell death-1 (PD-1) or programmed cell death-ligand 1 (PD-L1) inhibitions administrated after CRT. Clinical characteristics are presented in **Additional file 1: Table S2**. Approximately 8 – 10 ml of peripheral blood was collected at pre-treatment (at diagnosis) and post-CRT (1 month after CRT) landmark timepoints. Blood samples were centrifuged and processed within 2 h of collection to separate plasma from peripheral blood lymphocytes (PBLCs). Cell-free DNA (cfDNA) extracted from plasma was employed for library preparation and sequencing. Pre-treatment formalin-fixed paraffin-embedded (FFPE) tumor sections, if available, or baseline plasma combined with PBLCs (as a filter for clonal hematopoiesis) were applied for fixed-panel circulating tumor DNA (ctDNA) genotyping.

DNA extraction, library construction, and sequencing

The plasma was subjected to cfDNA extraction with a Qiagen QIAamp Circulating Nucleic Acid Kit (Qiagen, Dusseldorf, Germany). Purified cfDNA samples were qualified using Nanodrop2000 (Thermo Fisher Scientific, Waltham, MA, USA) and quantified using Qubit 2.0 dsDNA HS Assay Kit (Life Technologies, Waltham, MA, USA). Libraries were prepared using the KAPA Hyper Prep Kit (KAPA Biosystems, Wilmington, MA, USA). About 50 ng of cfDNA was sequentially underwent end-repairing, A-tailing, and ligation with indexed adapters, followed by size selection and polymerase chain reaction (PCR) amplification with KAPA Hyper DNA Library Prep Kit (KAPA Biosystems, Wilmington, MA, USA). Target enrichment was performed using customized xGen lockdown probes (Integrated DNA Technologies, Coralville, IA, USA) targeting 486 cancer-related genes (Nanjing Geneseeq Technology, Nanjing, China). Hybridization capture reaction was performed with

Dynabeads M-279 (Life Technologies, Waltham, MA, USA) and xGen Lockdown Hybridization and Wash Kit (Integrated DNA Technologies, Coralville, IA, USA) per manufacturers' instructions. Captured libraries were on-beads PCR amplified with Illumina p5 and p7 primers in KAPA HiFi HotStart ReadyMix (KAPA Biosystems, Wilmington, MA, USA), followed by purification using Agencourt AMPure XP beads. Libraries were quantified by quantitative PCR (qPCR) using KAPA Library Quantification kit (KAPA Biosystems, Wilmington, MA, USA). Library fragment size was determined by Bioanalyzer 2100 (Agilent Technologies, Santa Clara, CA, USA).

Next generation sequencing (NGS) and data processing

Sequencing was performed on the Illumina HiSeq4000 platform. Sequencing data were analyzed by Trimmomatic [1] to remove low-quality (quality < 15) or N bases, and then mapped to the human reference genome (hg19) using the Burrows-Wheeler Aligner. PCR duplicates were removed by Picard. The Genome Analysis Toolkit (GATK) was used to perform local realignments around indels and base quality reassurance. Single nucleotide polymorphisms (SNPs) and indels were analyzed by VarScan2 [2] and Haplotype Caller in GATK, with the mutant allele frequency cutoff of 0.2% for cfDNA samples, and a minimum of three unique mutant reads. Common SNPs were excluded if they were present in > 1% population frequency in the 1000 Genomes Project or the Exome Aggregation Consortium (ExAC) 65,000 exomes database. Gene fusions were identified by FACTERA [3]. The resulting mutation list was further filtered by an in-house list of recurrent artifacts based on a normal pool of whole blood samples. The raw sequencing data are deposited in the Genome Sequence Archive (GSA) for Human in National Genomics Data Center, under the accession numbers of HRA011737.

NCC-2 cohort

Study population and clinical procedures

NCC-2 cohort comprised 103 patients with resectable stage I – III NSCLC treated at NCC in Beijing, China from 2018 to 2022. All patients underwent curative-intent surgical resection. Clinical data are presented in **Additional file 1: Table S3**. Peripheral blood samples were collected at pre-treatment (before surgery) and post-treatment (1 month after surgery) timepoints. The procedures for peripheral blood collection and processing are consistent with the standard operating procedure (SOP) used in

the NCC-1 cohort. Surgically removed tumor tissue and pretreatment blood samples were subjected to fixed-panel ctDNA profiling.

DNA extraction, library construction, and sequencing

The plasma was separated by centrifugation at 3000× *g* for 10 min, and was subjected to cfDNA extraction with QIAamp Circulating Nucleic Acid Kit (Qiagen, Dusseldorf, Germany). FFPE tumor samples were de-paraffinized with xylene, and genomic DNA was extracted using QIAamp DNA FFPE Tissue Kit (Qiagen, Dusseldorf, Germany). Genomic DNA of the white blood cells were extracted using DNeasy Blood & Tissue kit (Qiagen, Dusseldorf, Germany). Purified genomic DNA was qualified using Nanodrop2000 (Thermo Fisher Scientific, Waltham, MA) and quantified using Qubit 3.0 dsDNA HS Assay Kit (Life Technologies, Waltham, MA, USA).

Libraries were prepared using the KAPA Hyper Prep Kit (KAPA Biosystems, Wilmington, MA, USA). For tumor tissue and control samples, 1 – 2 μg of genomic DNA underwent end-repairing, A-tailing and ligation with indexed sequencing adapters sequentially, followed by size selection using Agencourt AMPure XP beads (Beckman Coulter, Beverly, MA, USA). For plasma samples, about 50 ng of cfDNA underwent end-repairing, A-tailing, ligation with customized adapter containing unique molecular index (UMI), and PCR amplification with primers containing demultiplexing indices sequentially, followed by purification of cfDNA libraries using Agencourt AMPure XP beads (Beckman Coulter).

Different libraries with unique indices were pooled together in desirable ratios for up to 2 μg of total library input. Human cot-1 DNA (Life Technologies, Waltham, MA, USA) and xGen Universal blocking oligos (Integrated DNA Technologies, Coralville, IA, USA) were added as blocking reagents. Customized xGen lockdown probes (Integrated DNA Technologies, Coralville, IA, USA) targeting 139 lung cancer-relevant genes (Nanjing Geneseeq Technology, Nanjing, China) were used for hybridization enrichment. The capture reaction was performed with Dynabeads M-270 (Life Technologies, Waltham, MA, USA) and xGen Lockdown hybridization and wash kit (Integrated DNA Technologies, Coralville, IA, USA) per manufacturer's instructions. Captured libraries were on-beads PCR amplified with Illumina p5 and p7 primers in KAPA HiFi HotStart ReadyMix (KAPA Biosystems, Wilmington, MA, USA), followed by purification using Agencourt AMPure XP beads. Libraries were

quantified by qPCR using KAPA Library Quantification kit (KAPA Biosystems, Wilmington, MA, USA). Library fragment size was determined by Bioanalyzer 2100 (Agilent Technologies, Santa Clara, CA, USA). The target-enriched library was then sequenced on HiSeq4000 NGS platforms (Illumina, San Diego, CA, USA).

Mutation calling

Trimmomatic [1] was used for FASTQ file quality control, leading/trailing low quality (quality reading below 30) or N bases were removed. Qualified reads were then mapped to reference human genome (hg19) using Burrows-Wheeler Aligner [4]. PCR duplicates were removed by Picard (Broad Institute, MA, USA) after local realignment around known indels and base quality recalibration using GATK. For tissue specimens, single-nucleotide variations (SNVs) and insertion/deletion were detected using VarScan2[2] with default parameters. Genomic fusions were identified by FACTERA[3] with default parameters. Mutations that were observed in ≥ 20 cancer cases reported in the COSMIC database were defined as hotspots. A minimum variant allele frequency of 1% or 2% and minimum variant supporting reads of 5 or 6 were required for hotspot mutations or other mutations, respectively.

For cfDNA samples, single-stranded consensus sequences (SSCS) were generated by collecting all read pairs with the same mapping positions and grouping them into different SSCS families with the same UMI barcode sequences at both ends. Here, we required that a consensus read be supported by at least 2 reads. After the construction of the SSCS sequence, 2 SSCS read pairs with transposed UMI barcode sequences and the same mapping position were merged into 1 DCS, whenever possible. A local bioinformatics polishing pipeline was used to identify somatic variants in ctDNA after filtering out germline variants using normal control DNA. Mutations identified in the matched tumor DNA, which were supported by a minimum of one unique consensus mutant allele read and passed the polishing criteria were regarded as being present. ctDNA positivity was defined by assessing the presence of 1 or more mutations identified in the matched tumor sample in ctDNA. The raw sequencing data are deposited in the GSA-Human in National Genomics Data Center, under the accession numbers of HRA001346.

Stanford cohort

Stanford cohort involved 37 patients from 2 observational studies (NCT01385722, NCT00349830) treated at Stanford University, California, USA, from 2010 to 2016. All samples were collected with informed consent and institutional review board approval in accordance with the Declaration of Helsinki [5]. All patients had localized stage I – III diseases and underwent curative-intent treatments, including surgery or definitive CRT (**Additional file 1: Table S4**). Blood samples were collected at pre-treatment (at diagnosis) and post-treatment (within 4 months after completing radical treatment) timepoints. Tumor genotyping was performed with cancer personalized profiling by deep sequencing (CAPP-seq) fixed panel based on tumor tissue or pretreatment blood specimens (plasma and plasma-depleted whole blood) [5, 6].

LUCID cohort

LUCID (LUng cancer Circulating tumour DNA) study cohort included 100 patients with stage I – III NSCLC treated at Royal Papworth Hospital or Addenbrooke's Hospital in Cambridge, UK (NCT04153526). The LUCID study was approved by the local research ethics committee (REC: 14/WM/1072) [7]. Eligible subjects were treated with radical surgery or CRT (**Additional file 1: Table S5**). Peripheral blood samples were collected at pre-treatment (baseline) and post-treatment (within 2 weeks to 4 months after treatment end) landmark timepoints. FFPE tumor tissue samples obtained from surgical resection or diagnostic biopsies were used for whole-exome sequencing (WES) to identify tumor-specific variants for the design of patient-specific panels. Plasma was applied to extract cfDNA. Buffy coat samples, which contained the layer of PBMCs, were isolated for DNA extraction. Personalized ctDNA panels (RaDaR^{TM}) [7] were crafted by ranking and prioritizing buffy coat DNA, with a focus on 48 amplicons per individual to target patient-specific variants.

TRACERx cohort

A total of 100 patients from TRACERx (Tracking Non-Small-Cell Lung Cancer Evolution Through Therapy), a prospective observational multicenter cohort study conducted in UK (NCT01888601), were included. The TRACERx study was approved by an independent Research Ethics Committee (13/LO/1546) [8]. Treatment-naïve patients with resectable stage I – III NSCLC were eligible and underwent radical surgery (**Additional file 1: Table S6**). Peripheral blood specimens were collected

at pre- and post-surgical timepoints. WES was performed on DNA purified from tumor and blood samples. A customized multiplex-PCR NGS approach (ArcherDX) was utilized for personalized ctDNA profiling panels, which included design of specific multiplex-PCR primers for each patient targeting somatic single nucleotide variants detected in tumor samples, and amplifying ctDNA in peripheral blood through multiplex-PCR to characterize genomic features [8, 9]. This cohort was employed for independent validation.

Moding et al. cohort

Moding et al. cohort comprised 48 patients from a phase II trial (NCT02525757) and 2 observational studies (MDACC-LAB09-0983, NCT00349830) at University of Texas MD Anderson Cancer Center and Stanford University in USA [10]. All samples were collected with informed consent in accordance with the Declaration of Helsinki and approved by the Institutional Review Board (33868) [10]. Patients with unresectable stage II – III NSCLC receiving CRT or CRT followed by consolidation ICI were eligible (**Additional file 1: Table S7**) and collected blood samples for ctDNA analysis at pre-treatment and post-CRT (within 4 months after CRT) timepoints. Pre-treatment FFPE tumor sections, if available, were employed to isolate tumor DNA, and PBLCs were used for germline DNA isolation. Genomic DNA obtained from PBLCs and tumor tissues was fragmented before library preparation. cfDNA was extracted from plasma for ctDNA library preparation and sequencing. Overall, tumor, plasma, and PBLCs samples were analyzed by fixed-panel CAPP-seq platform to profile genomic alterations [10]. This cohort was used for independent validation.

References

1. Bolger AM, Lohse M, Usadel B. Trimmomatic: a flexible trimmer for Illumina sequence data. Bioinformatics. 2014;30(15):2114-20.
2. Koboldt DC, Zhang Q, Larson DE, Shen D, McLellan MD, Lin L, et al. VarScan 2: somatic mutation and copy number alteration discovery in cancer by exome sequencing. Genome Res. 2012;22(3):568-76.
3. Newman AM, Bratman SV, Stehr H, Lee LJ, Liu CL, Diehn M, et al. FACTERA: a practical method for the discovery of genomic rearrangements at breakpoint resolution. Bioinformatics. 2014;30(23):3390-3.
4. Li H, Durbin R. Fast and accurate short read alignment with Burrows-Wheeler transform. Bioinformatics. 2009;25(14):1754-60.
5. Chaudhuri AA, Chabon JJ, Lovejoy AF, Newman AM, Stehr H, Azad TD, et al. Early detection of molecular residual disease in localized lung cancer by circulating tumor DNA profiling. Cancer Discov. 2017;7(12):1394-403.
6. Newman AM, Lovejoy AF, Klass DM, Kurtz DM, Chabon JJ, Scherer F, et al. Integrated digital error suppression for improved detection of circulating tumor DNA. Nat Biotechnol. 2016;34(5):547-55.
7. Gale D, Heider K, Ruiz-Valdepenas A, Hackinger S, Perry M, Marsico G, et al. Residual ctDNA after treatment predicts early relapse in patients with early-stage non-small cell lung cancer. Ann Oncol. 2022;33(5):500-10.
8. Abbosh C, Birkbak NJ, Wilson GA, Jamal-Hanjani M, Constantin T, Salari R, et al. Phylogenetic ctDNA analysis depicts early-stage lung cancer evolution. Nature. 2017;545(7655):446-51.
9. Jamal-Hanjani M, Wilson GA, McGranahan N, Birkbak NJ, Watkins TBK, Veeriah S, et al. Tracking the evolution of non-small-cell lung cancer. N Engl J Med. 2017;376(22):2109-21.
10. Moding EJ, Liu Y, Nabet BY, Chabon JJ, Chaudhuri AA, Hui AB, et al. Circulating tumor DNA dynamics predict benefit from consolidation immunotherapy in locally advanced non-small cell lung cancer. Nat Cancer. 2020;1(2):176-83.

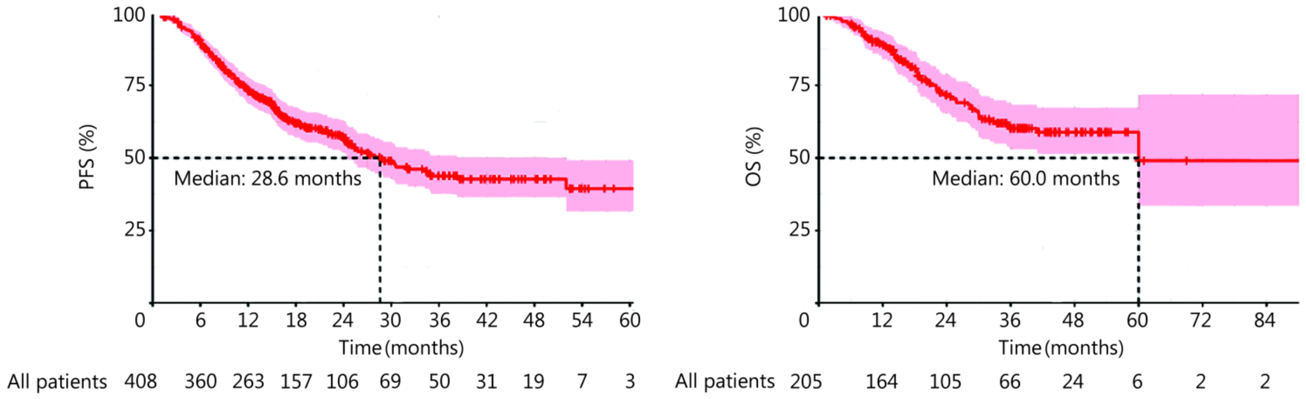

Fig. S1 Survival outcomes. Kaplan-Meier curve of progression-free survival (PFS) and overall (OS) survival for all patients

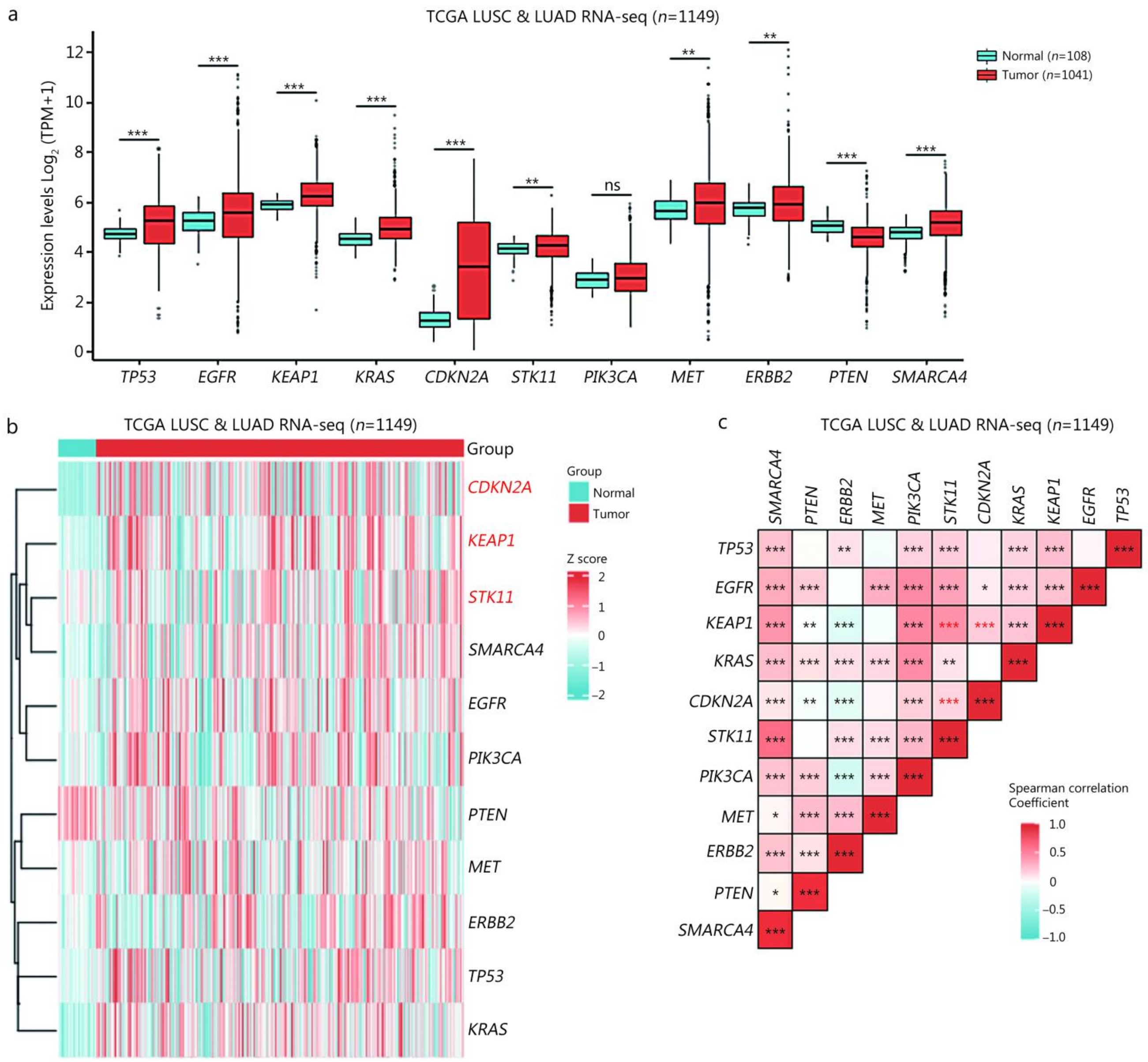

Fig. S2 RNA-sequencing (RNA-seq) analysis of *KEAP1*, *STK11*, and *CDKN2A* mutations in the Cancer Genome Atlas (TCGA) database. **a** Box plots comparing the expression levels of selected genes in normal and tumor samples. **b** Heatmap displaying the clustered gene expression profiles in NSCLC compared to normal controls. *KEAP1*, *STK11*, and *CDKN2A* are denoted in red. **c** Heatmap of Spearman correlations. Colors represent correlation coefficients. The correlation coefficients between *CDKN2A* and *KEAP1*, and between *CDKN2A* and *STK11*, were lower than that between *KEAP1* and *STK11*. $^{**}P < 0.01$, $^{***}P < 0.001$, ns not significant. TCGA The Cancer Genome Atlas, LUSC lung squamous cell carcinoma, LUAD lung adenocarcinoma, RNA-seq RNA-sequencing, TPM transcripts per million

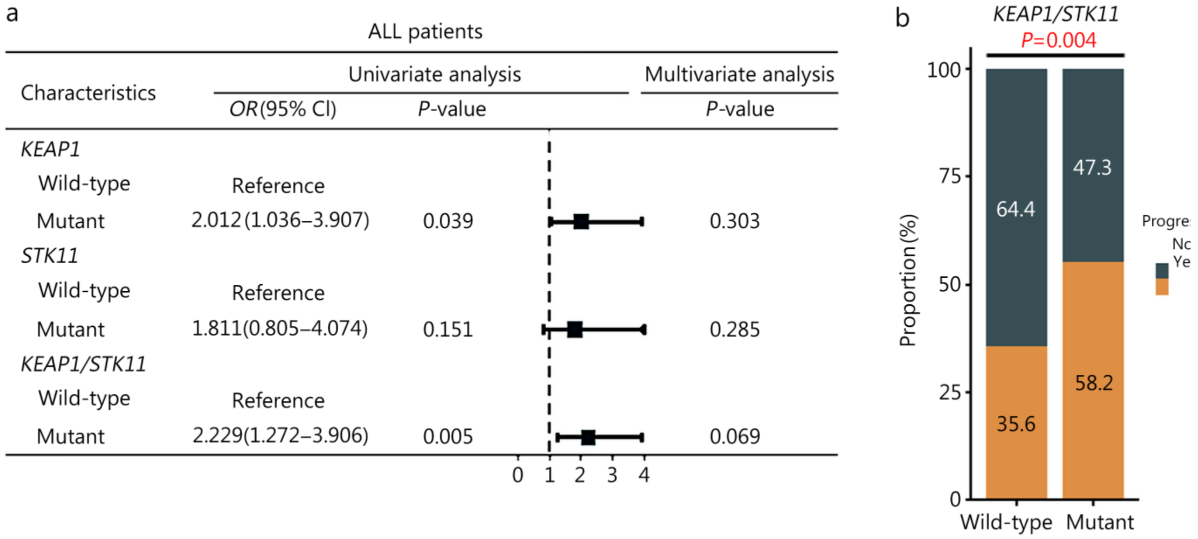

Fig. S3 Prognostic effects of *KEAP1/STK11* mutations in the overall patient cohort. **a** Univariate and multivariate logistic regression analyses of *KEAP1*, *STK11*, and combined *KEAP1/STK11* mutations on disease progression in all patients. **b** Proportion of disease progression in *KEAP1/STK11* wildtype vs. mutant patients. *OR* odds ratio, CI confidence interval

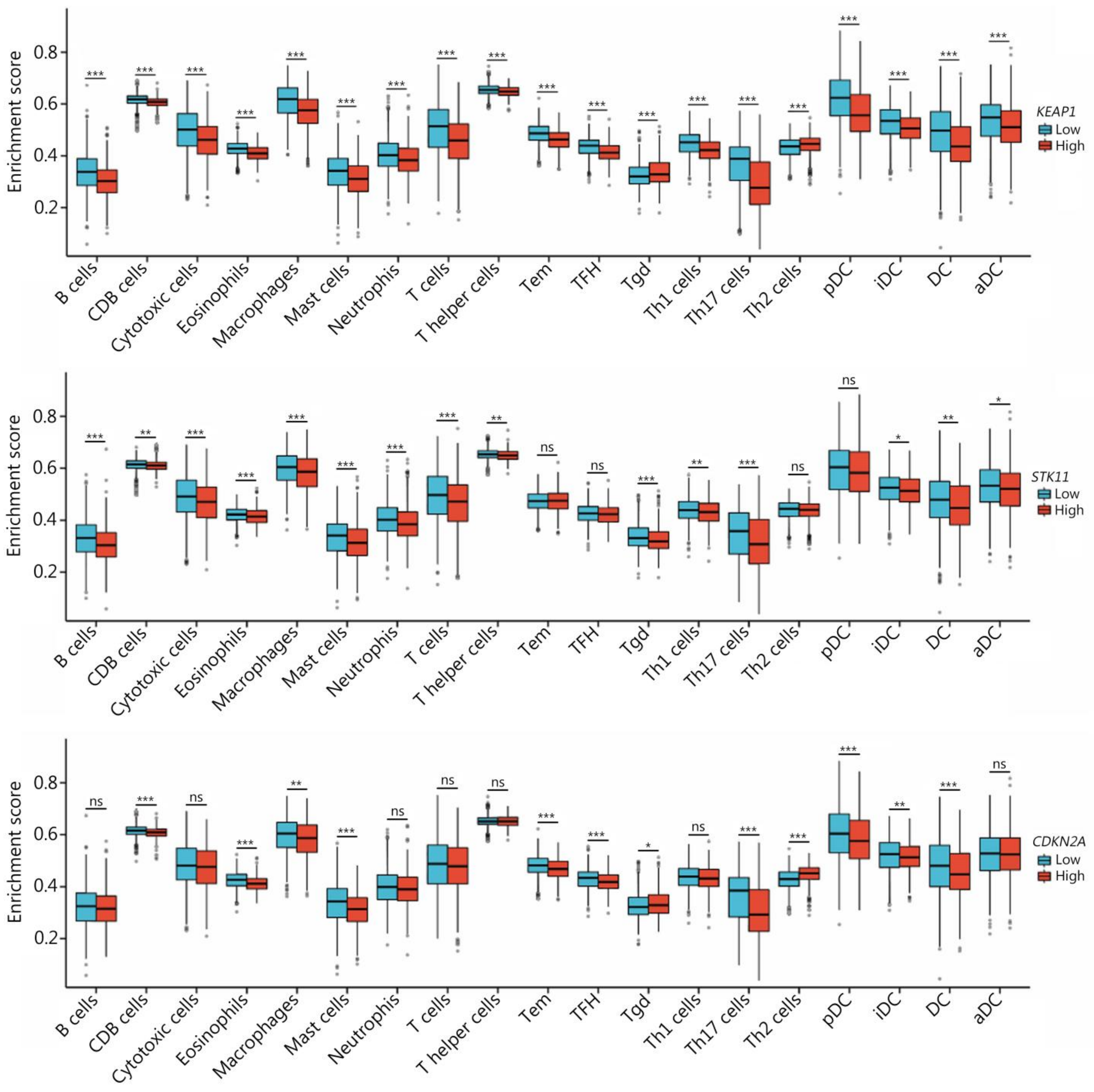

Fig. S4 Immune infiltration analysis of *KEAP1/STK11/CDKN2A* mutations. Box plots depicting significant decreases of various immune cells in the tumor microenvironment of patients with high *KEAP1, STK11* and *CDKN2A* expressions in TCGA. $^{*}P < 0.05$, $^{**}P < 0.01$, $^{***}P < 0.001$, ns not significant. Tem T effector memory, TFH T follicular helper, Tgd T gamma delta, Th T helper, pDC plasmacytoid dendritic cell, iDC immature dendritic cell, DC dendritic cell, aDC activated dendritic cell

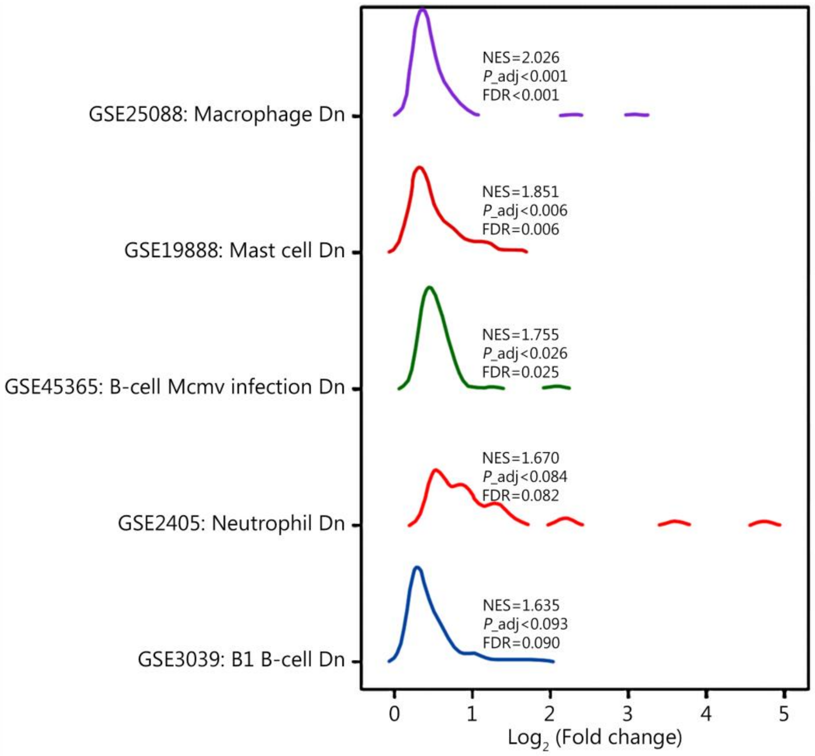

Fig. S5 Gene set enrichment analysis (GSEA) ridge plots showing the normalized enrichment score (NES), adjusted *P*-value (*P*_adj), and false discovery rate (FDR) for significantly enriched gene sets. Dn downregulated, Mcmv murine cytomegalovirus

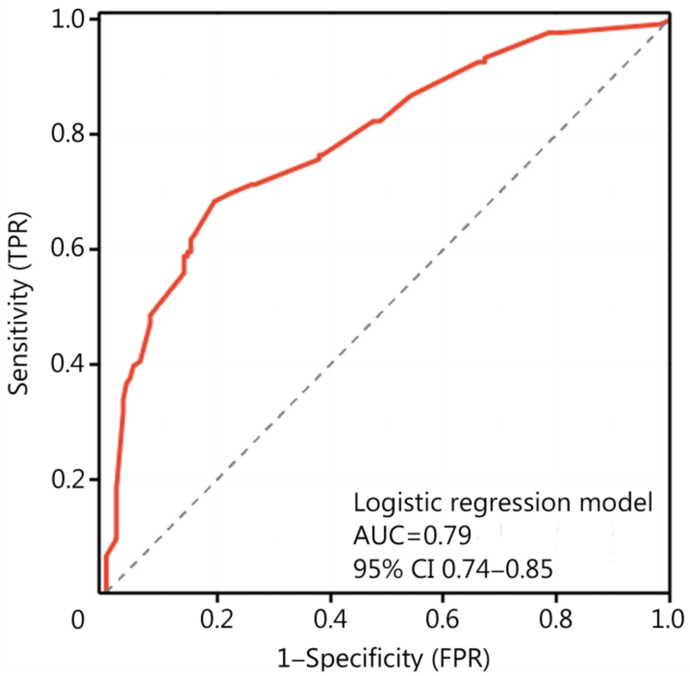

Fig. S6 Receiver operating characteristic (ROC) curve for the combined model based on logistic regression. TPR true positive rate, AUC area under the curve, CI confidence interval, FPR false positive rate

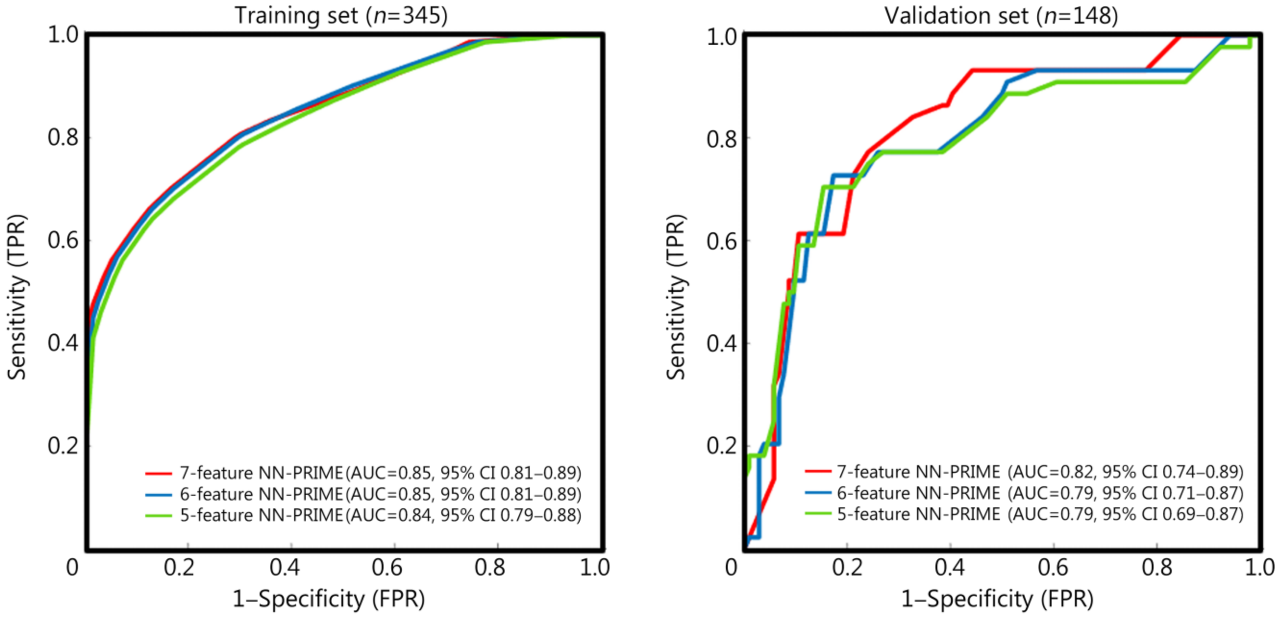

Fig. S7 Comparison of model performance with composite vs. separate genomic features. Receiver operating characteristic (ROC) curves for 7-feature model (based on mutations in *KEAP1*, *STK11*, and *CDKN2A*, clinical stage, treatment modality, pre-treatment ctDNA, and post-treatment MRD), 6-feature model (based on *KEAP1/STK11*, *CDKN2A* and other features), and 5-feature model (*KEAP1/STK11/CDKN2A* and other features) in the training and validation sets. TPR true positive rate, NN neural network, AUC area under the curve, CI confidence interval, FPR false positive rate

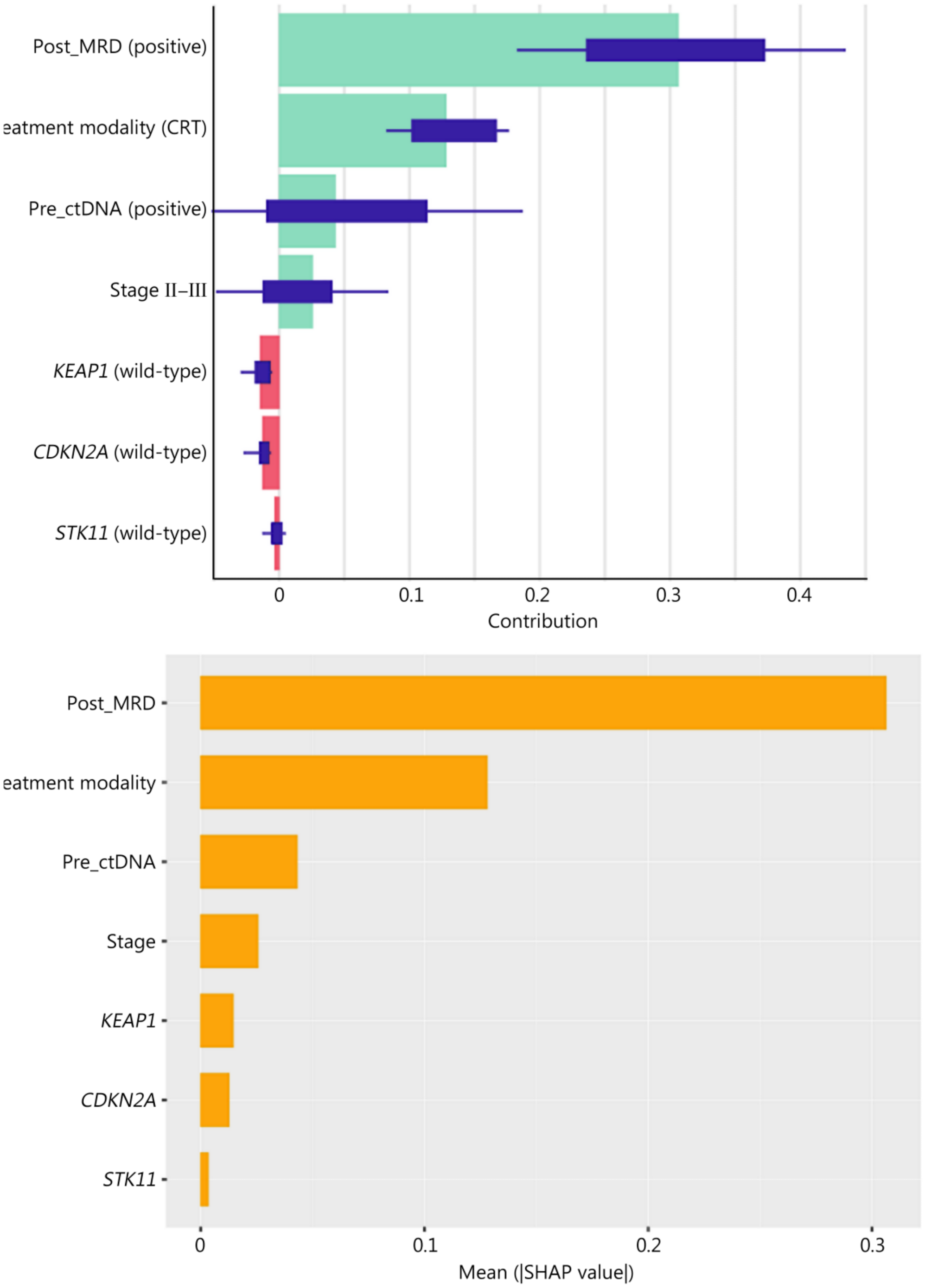

Fig. S8 SHapley Additive exPlanations (SHAP) for neural network (NN)-based PRIME model. **a** Rank of each feature according to its contribution to the model prediction. **b** Rank of each feature according to SHAP value. Post_MRD post-treatment minimal residual disease, CRT chemoradiotherapy, Pre_ctDNA pre-treatment circulating tumor DNA

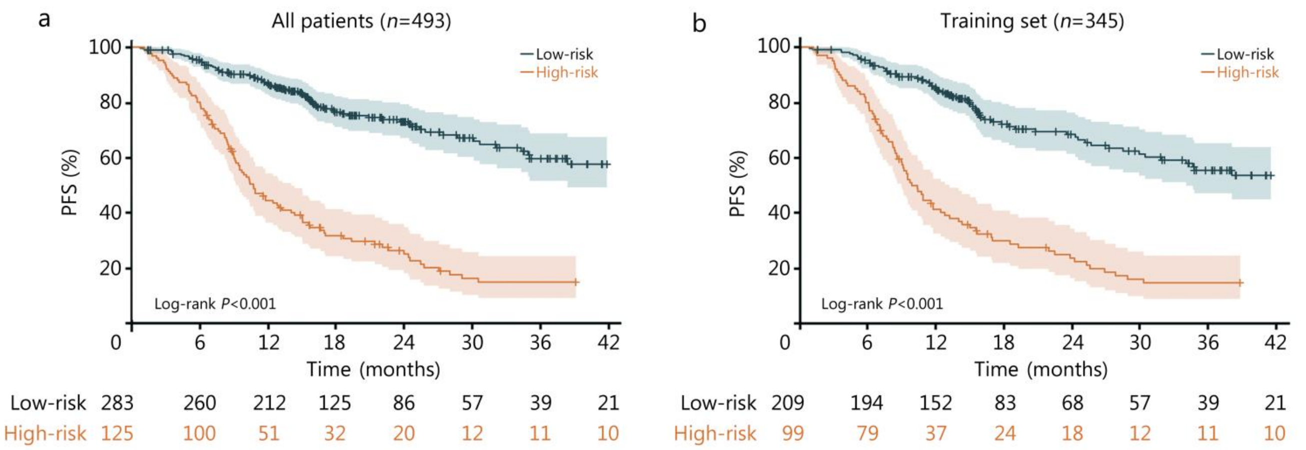

Fig. S9 Progression-free survival (PFS) stratified by NN-PRIME in all patients (**a**) and in the training set (**b**). NN neural network, PRIME Progression Risk prediction by Interpretable Machine learning on ctDNA-MRD, Mutations, and clinical-therapeutic features

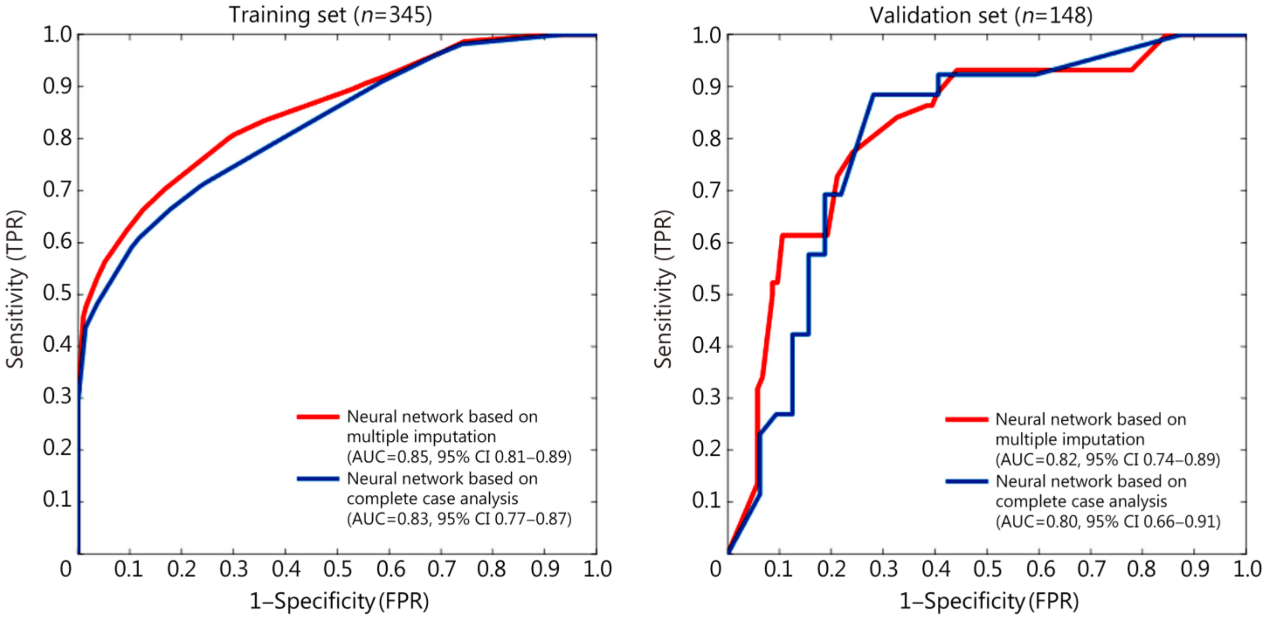

Fig. S10 Sensitivity analyses of NN-PRIME model robustness. Receiver operating characteristic (ROC) curves of the NN-PRIME model based on multiple imputation vs. based on complete-case analysis in the training and validation sets. TPR true positive rate, AUC area under the curve, CI confidence interval, FPR false positive rate, NN neural network, PRIME Progression Risk prediction by Interpretable Machine learning on ctDNA-MRD, Mutations, and clinical-therapeutic features

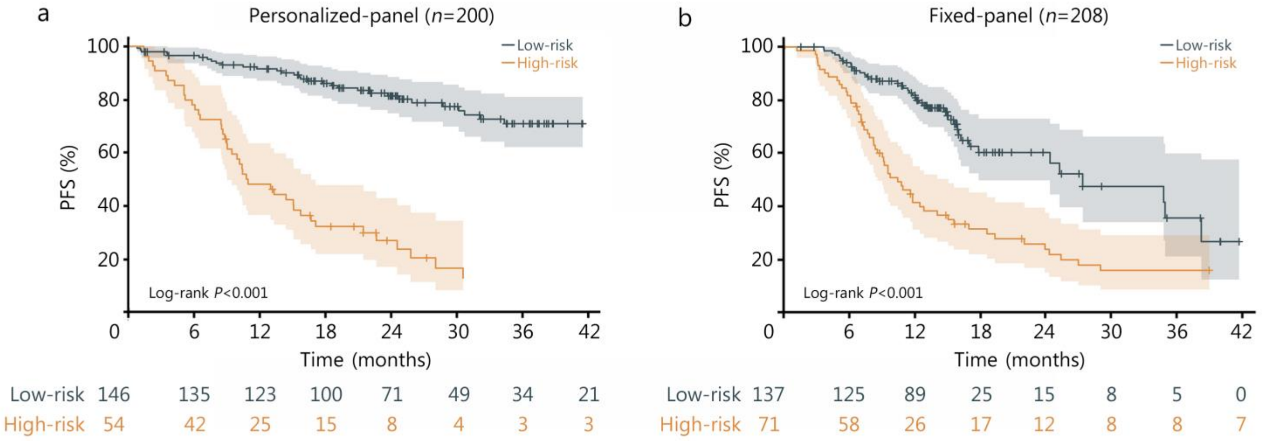

Fig. S11 Progression-free survival (PFS) stratified by NN-PRIME in patients receiving personalized-panel (tumor-informed) ctDNA testing (**a**) and in patients receiving fixed-panel (tumor-naïve) ctDNA testing (**b**). Among all patients who underwent fixed-panel ctDNA testing (n = 293), 85 were excluded from the analysis due to the lack of time-to-event data. NN neural network, PRIME Progression Risk prediction by Interpretable Machine learning on ctDNA-MRD, Mutations, and clinical-therapeutic features

Variables	*N* (%)	Low-risk (events/total)	High-risk (events/total)	*OR* (95% CI)	*P*-value
All patients	493(100.0)	82/333	128/160	0.08(0.05–0.13)	<0.001
ctDNA sequencing platform					
ArcherDX	100(20.3)	9/74	17/26	0.07(0.03–0.21)	<0.001
CAPP-seq	85(17.2)	8/50	30/35	0.03(0.01–0.11)	<0.001
Geneseeq Tech	208(42.2)	43/137	56/71	0.12(0.06–0.24)	<0.001
RaDaR™	100(20.3)	22/72	25/28	0.05(0.01–0.19)	<0.001
ctDNA detection technique					
Fixed-panel detection	293(59.4)	51/187	86/106	0.09(0.05–0.16)	<0.001
Personalized-panel detection	200(40.6)	31/146	42/54	0.08(0.04–0.16)	<0.001
Cohort (platform)					
NCC-1 cohort (Geneseeq Tech)	105(21.3)	24/58	38/47	0.17(0.07–0.41)	<0.001
NCC-2 cohort (Geneseeq Tech)	103(20.9)	19/79	18/24	0.11(0.04–0.30)	<0.001
Stanford cohort (CAPP-seq)	37(7.5)	0/14	20/23	0(0–Inf)	>0.990
LUCID cohort (RaDaR™)	100(20.3)	22/72	25/28	0.05(0.01–0.19)	<0.001
TRACERx cohort (ArcherDX)	100(20.3)	9/74	17/26	0.07(0.03–0.21)	<0.001
Moding et al. cohort (CAPP-seq)	48(9.7)	8/36	10/12	0.06(0.01–0.32)	0.001

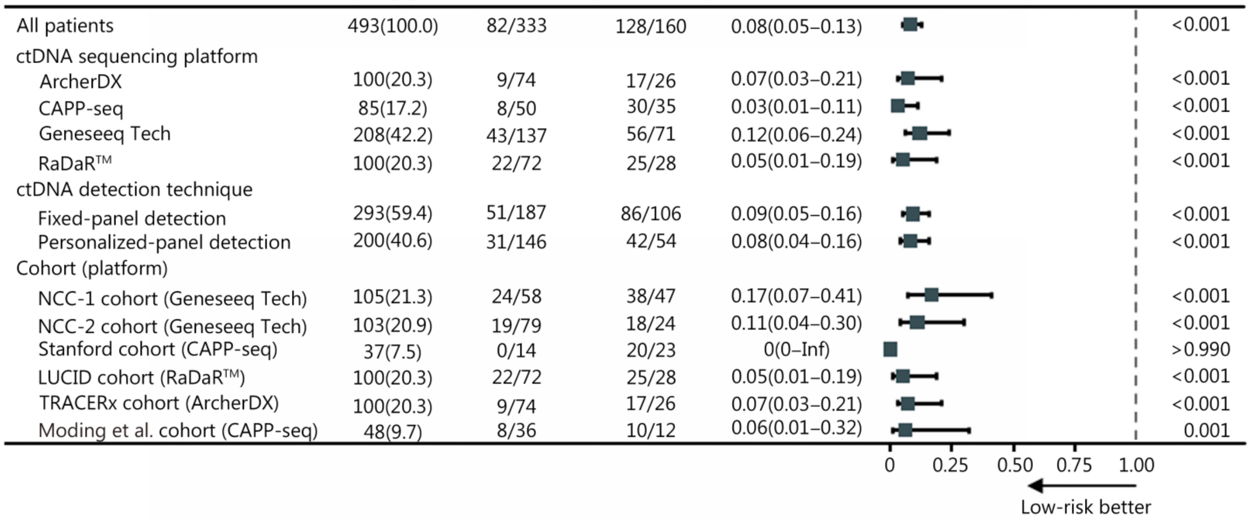

Fig. S12 Subgroup analyses of NN-PRIME model performance in different ctDNA sequencing platforms, detection techniques, and patient cohorts. *OR* odds ratio, CI confidence interval, ctDNA circulating tumor DNA, CAPP-seq cancer personalized profiling by deep sequencing, NCC National Cancer Center, LUCID LUng cancer Circulating tumour DNA, TRACERx Tracking Non-Small-Cell Lung Cancer Evolution Through Therapy, NN neural network, PRIME Progression Risk prediction by Interpretable Machine learning on ctDNA-MRD, Mutations, and clinical-therapeutic features, Inf infinite

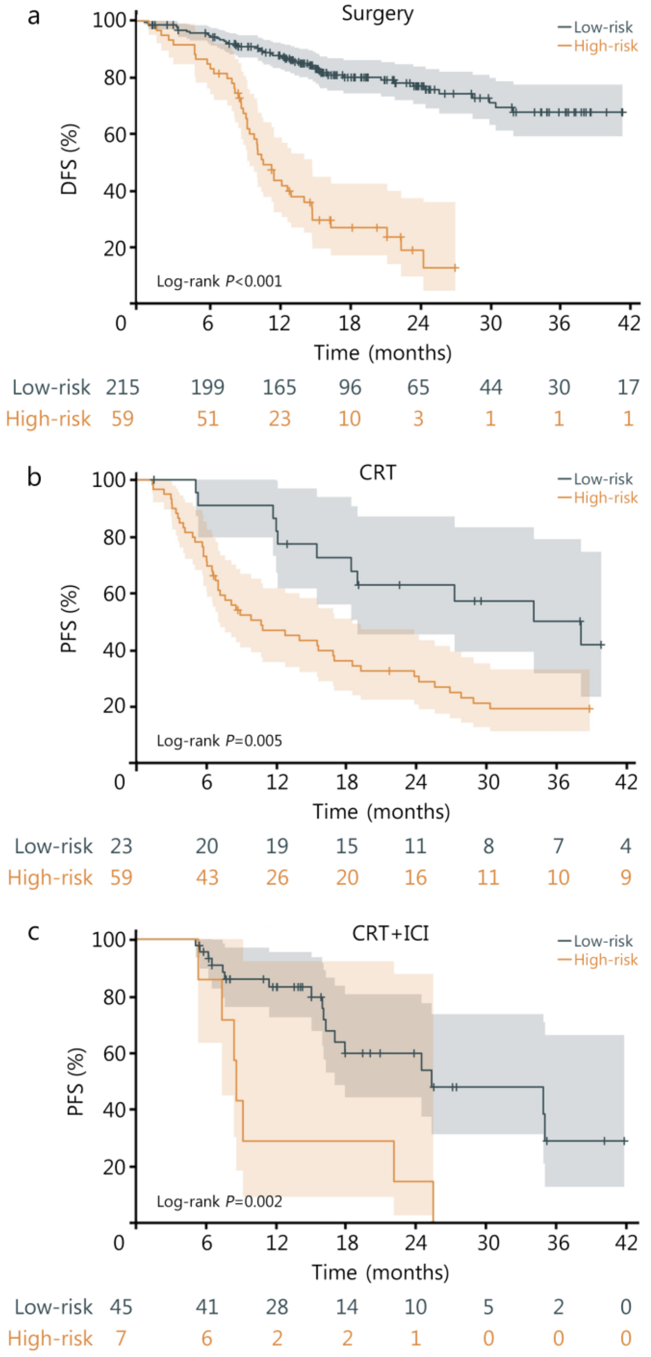

Fig. S13 Kaplan-Meier curves of survival outcomes in patients with different treatment regimens. **a** Disease-free survival (DFS) stratified by NN-PRIME in patients receiving surgery. Progression-free survival (PFS) stratified by NN-PRIME in patients receiving definitive chemoradiotherapy (CRT) alone (**b**) and in patients with CRT and consolidation immune checkpoint inhibitor (ICI) therapy (**c**). NN neural network, PRIME Progression Risk prediction by Interpretable Machine learning on ctDNA-MRD, Mutations, and clinical-therapeutic features

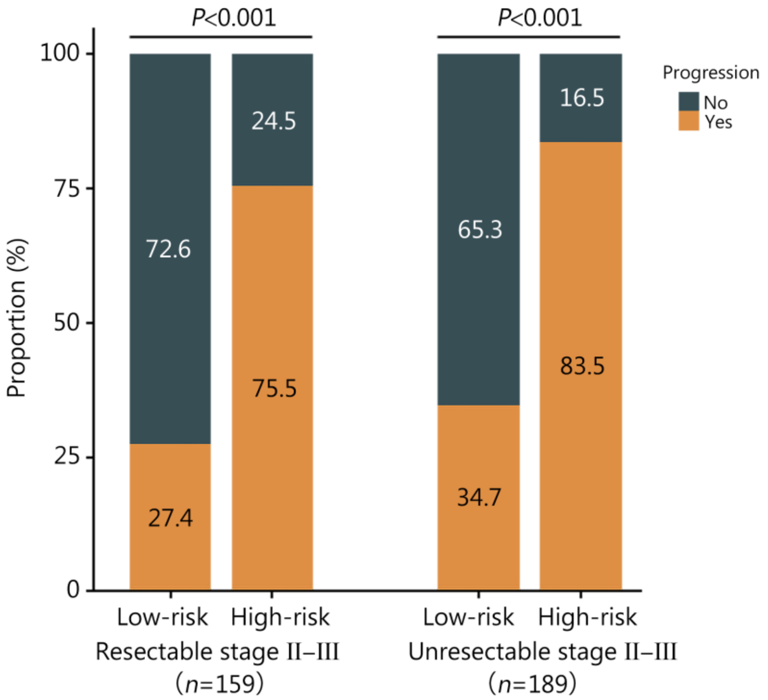

Fig. S14 Proportion of disease progression in low-risk vs. high-risk patients stratified by NN-PRIME in resectable or unresectable stage II – III NSCLC patients. NN neural network, PRIME Progression Risk prediction by Interpretable Machine learning on ctDNA-MRD, Mutations, and clinical-therapeutic features

Variables	*N* (%)	High-risk (events/total)	Low-risk (events/total)	*OR* (95% CI)	*P*-value	*P*-value for interaction
All patients	493(100.0)	126/160	70/333	0.07(0.05–0.11)	<0.001	
Age						0.901
≤59 years	73(14.8)	24/31	9/42	0.08(0.03–0.24)	<0.001	
60–69 years	138(28.0)	41/52	18/86	0.07(0.03–0.17)	<0.001	
70–74 years	49(9.9)	12/16	6/33	0.07(0.02–0.31)	<0.001	
≥75 years	82(16.6)	21/25	10/57	0.04(0.01–0.14)	<0.001	
NA	151(30.6)	28/36	27/115	0.09(0.04–0.21)	<0.001	
Sex						0.450
Female	117(23.7)	27/31	17/86	0.04(0.01–0.12)	<0.001	
Male	225(45.6)	71/93	26/132	0.08(0.04–0.14)	<0.001	
NA	151(30.6)	28/36	27/115	0.09(0.04–0.21)	<0.001	
Smoking						0.808
NA	14(2.8)	4/5	1/9	0.03(0.01–0.64)	0.025	
No	101(20.5)	18/22	22/79	0.09(0.03–0.28)	<0.001	
Yes	378(76.7)	104/133	47/245	0.07(0.04–0.11)	<0.001	
ECOG						0.044
0	73(14.8)	13/18	12/55	0.11(0.03–0.36)	<0.001	
1	127(25.8)	35/50	20/77	0.15(0.07–0.33)	<0.001	
NA	293(59.4)	78/92	38/201	0.04(0.02–0.08)	<0.001	
Histology						0.484
Adeno	257(52.1)	61/75	37/182	0.06(0.03–0.12)	<0.001	
NA	17(3.5)	5/7	1/10	0.04(0.01–0.62)	0.021	
Other	30(6.1)	13/14	4/16	0.03(0.01–0.26)	0.002	
SCC	189(38.3)	47/64	28/125	0.10(0.05–0.21)	<0.001	
Stage						0.773
I	130(26.4)	9/12	11/118	0.03(0.01–0.15)	<0.001	
II	111(22.5)	26/37	12/74	0.08(0.03–0.21)	<0.001	
IIIA	124(25.2)	46/55	25/69	0.11(0.05–0.26)	<0.001	
IIIB	92(18.7)	34/42	17/50	0.12(0.05–0.32)	<0.001	
IIIC	21(4.3)	8/10	3/11	0.09(0.01–0.72)	0.023	
NA	15(3.0)	3/4	2/11	0.07(0.00–1.14)	0.062	
Treatment						0.994
CRT	134(27.2)	74/91	10/43	0.07(0.03–0.17)	<0.001	
CRT+ICI	80(16.2)	8/9	24/71	0.06(0.01–0.54)	0.012	
Surgery	279(56.6)	44/60	36/219	0.07(0.04–0.14)	<0.001	
Pre_ctDNA						0.132
Negative	158(32.1)	21/36	21/122	0.15(0.07–0.33)	<0.001	
Positive	281(57.0)	89/105	43/176	0.06(0.03–0.11)	<0.001	
NA	54(11.0)	16/19	6/35	0.04(0.01–0.18)	<0.001	
Post_MRD						0.045
Negative	224(45.4)	19/29	45/195	0.16(0.07–0.36)	<0.001	
Positive	118(23.9)	78/89	16/29	0.17(0.07–0.46)	<0.001	
NA	151(30.6)	29/42	9/109	0.04(0.02–0.10)	<0.001	
Dataset						0.958
Training	345(70.0)	99/122	53/223	0.07(0.04–0.13)	<0.001	
Validation	148(30.0)	27/38	17/110	0.07(0.03–0.18)	<0.001	

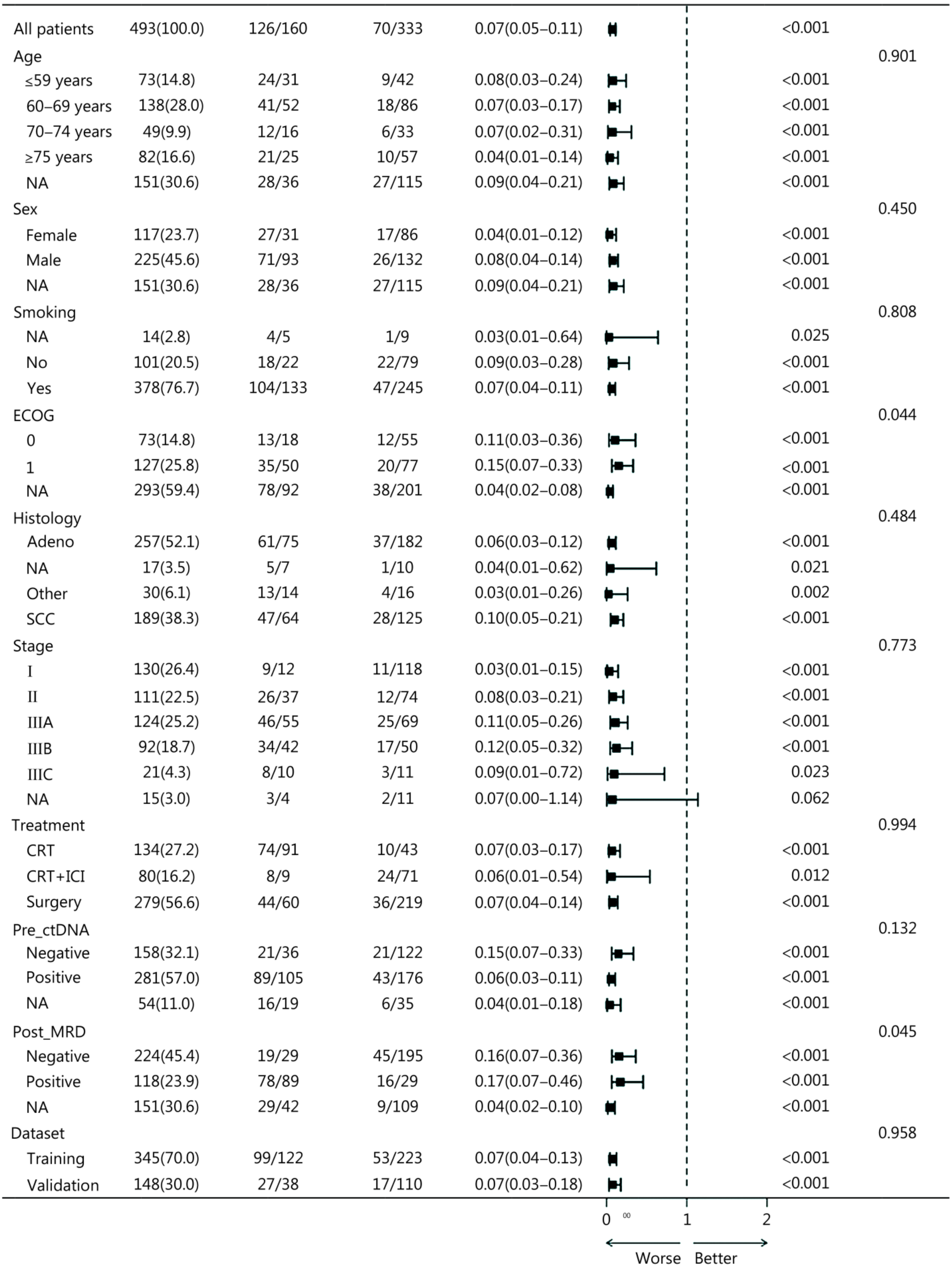

Fig. S15 Forest plot of logistic regression indicating high-risk patients identified by NN-PRIME correlated with poorer outcomes across various clinical subgroups. *OR* odds ratio, CI confidence

interval, NA not available, ECOG Eastern Cooperative Oncology Group, Adeno adenocarcinoma, SCC squamous cell carcinoma, CRT chemoradiotherapy, ICI immune checkpoint inhibitor, Pre_ctDNA pre-treatment circulating tumor DNA, Post_MRD post-treatment minimal residual disease, NN neural network, PRIME Progression Risk prediction by Interpretable Machine learning on ctDNA-MRD, Mutations, and clinical-therapeutic features
